# Neural network representations of multiphase Equations of State

**DOI:** 10.1038/s41598-024-81445-4

**Published:** 2024-12-05

**Authors:** George A. Kevrekidis, Daniel A. Serino, M. Alexander R. Kaltenborn, J. Tinka Gammel, Joshua W. Burby, Marc L. Klasky

**Affiliations:** 1 Los Alamos National Laboratory, Los Alamos, NM USA; 2 Department of Applied Mathematics and Statistics, Johns Hopkins University, Baltimore, MD USA; 3 Department of Physics, University of Texas at Austin, Austin, TX USA

**Keywords:** Materials science, Physics, Chemistry, Physical chemistry, Mathematics and computing, Applied mathematics

## Abstract

Equations of State model relations between thermodynamic variables and are ubiquitous in scientific modelling, appearing in modern day applications ranging from Astrophysics to Climate Science. The three desired properties of a general Equation of State model are adherence to the Laws of Thermodynamics, incorporation of phase transitions, and multiscale accuracy. Analytic models that adhere to all three are hard to develop and cumbersome to work with, often resulting in sacrificing one of these elements for the sake of efficiency. In this work, two deep-learning methods are proposed that provably satisfy the first and second conditions on a large-enough region of thermodynamic variable space. The first is based on learning the generating function (thermodynamic potential) while the second is based on structure-preserving, symplectic neural networks, respectively allowing modifications near or on phase transition regions. They can be used either “from scratch” to learn a full Equation of State, or in conjunction with a pre-existing consistent model, functioning as a modification that better adheres to experimental data. We formulate the theory and provide several computational examples to justify both approaches, highlighting their advantages and shortcomings.

## Introduction

Equations of State (EoSs) are ubiquitous in scientific modelling and have been studied extensively for the better part of the past two centuries, as they are essential in a wide variety of fields including astrophysics (e.g.^1–6^), climate research (e.g.^7–9^), hydrodynamics (e.g.^10^), plasma models in nuclear fusion (e.g.^11^), and asteroid impact modelling (e.g.^12^), among others. In their most elementary form, EoS models consist of closed-form equations, such as the ideal gas law^13^, Van der Waals^14^, and those derived from statistical mechanics e.g., the Virial equation^15,16^. These analytic models are usually not universally accurate across many elements or even different phases of a single element.

### Background

Physical properties of fluids can, and have, been measured experimentally since the dawn of modern science. These properties include densities, vapor pressures, critical point data, viscosities, solubilities, surface tensions, thermal conductivities, etc., and are collated in existing open-access and closed-source databases^17^. However, the cost of experimental acquisition of the data is at odds with the massive phase space for both pure and mixture systems of interest. Consequently, there has been a sustained effort to establish empirical relationships characterizing the EoS, enabling the determination of relationships among state variables such as density, pressure, and temperature. Indeed, the transformative work of Van der Waals provided insights into the pressure-volume temperature relationship for both liquids and gases and the interpretation of a critical condition that limited the coexistence of these phases. Using a theoretical procedure, Van der Waals derived a simple mathematical function, a third degree polynomial, capable of fitting experimental data for simple fluids. This model has guided a century of research into finding the ultimate mathematical function that can relate the macroscopic thermodynamic properties of fluids to each other^18,19^.

Over the next century, the development of a more general theory connecting molecular model parameters with macroscopic observables emerged, driven by the recognition of matter’s molecular nature and advances in statistical mechanics^19^. While these simplified models have proven useful, they are limited by various issues hindering accurate EoS characterization across the extensive phase space typically encountered^20^. Indeed, the development of an EoS has been marked by a long and convoluted search, frequently yielding unsatisfactory outcomes, underscoring the absence of a universally accepted EoS, even for the simplest fluids^18,19^. Consequently, the majority of high-pressure EoS are empirical in nature and rely on limited sets of experimental data to reproduce the relevant physics^18^. Examples of such models include the Jones Wilkens Lee (JWL), Davis reactants, Mie-Grüneisen, and Tillotson EoSs^21–26^. Many of these models are based on a reference curve that can be a Hugoniot, an isentrope, or an isotherm. These forms in general allow for simple linear extrapolation and are fairly accurate when the desired properties are in close proximity to the reference curve. However, significant deviations from empirical reality arise when attempting to move away from the reference curve^24,25,27,28^.

Current state-of-the-art methods such as SESAME, ANEOS, and HerEOS^10,29–31^ produce ‘global’, multi-phase EoS models. They achieve this by representing thermodynamic variables with (often analytic) techniques across different scales and phases to interpolate among predefined grid points. This approach aims to create a ‘complete’ thermodynamic model. However, the discrete values and their distribution might be less than ideal, often subject to interpolation errors. These errors can disrupt thermodynamic consistence and negatively affect calculations dependent on these properties^10^. Furthermore, efficient processing of the full range of a multi-phase table requires logarithmic sampling of the phase space, which may not provide sufficient resolution to identify phase transitions from the tabular data, also requiring additional information to be provided to the model. Nevertheless, these EoS models have been utilized for the last several decades. A general review of present challenges with state-of-the-art EoS models is available, see^32^.

### Relevant literature

The emergence of machine learning has given promise to a new approach for building multi-phase EoS tables for downstream applications. Indeed, recent investigations have focused on addressing the question as to whether ML methods may be designed to substitute for analytical models^17,19,20,33^. In this regard, Arce et al. compared the quality of fit for common EoS binary systems involving solvents and bio-diesel components at supercritical conditions, finding good agreement among the EoS, ML model, and experimental data^33^. In a separate study, researchers explored the potential of using ML to correlate extensive datasets of physical properties. These datasets were produced using cutting-edge analytical EoS, specifically the Statistical Associating Theory variable range Mie equation (SAFT-VR-Mie) EoS for pure fluids^17^. Further research has been conducted on utilizing ML to develop EoSs that *exactly* adhere to energy conservation principles. This research combines these EoS with the robust, expressive power of black- or grey-box ML methods^19,20^. In these, the general approach centers around estimating a smooth energy potential (e.g., Helmholtz free energy), and differentiating it, through automatic differentiation, to generate the remaining relevant thermodynamic quantities. The advantage of this approach is that the model is exactly consistent with thermodynamics. The model has been tested for the van der Waals, Lennard-Jones fluids, as well as Mie fluids^19,20^.

The latter approach is outlined in “The (additive) graph correction” section. However, near phase transitions one may encounter difficulty using a smooth neural network since the approximation will not be consistent (Example 3.1). Using non-smooth activation functions, such as ReLU, would give a network the expressivity needed to model the transitions; however, it is exceedingly hard to control their ‘location’ in phase space numerically, so the right number and topology of phase transitions using such a model would not be guaranteed, at least without further structural modifications of the architectures. Initial attempts to address this issue have been made using multiple networks that are used for different phases (or scales) of an EoS^34^. While this allows for a model to perform well in the multiscale setting that is natural to the problem at hand, one cannot guarantee that ‘gluing’ these models around phase transitions *is* thermodynamically consistent. This is a fundamental limitation to global EoS modelling with neural networks, which are known to not perform well in wide, multiscale settings^35^.

In summary, it is technically difficult to develop neural EoS models that simultaneously combine thermodynamic consistence, multi-scale accuracy, and robust handling of phase transitions, which are all desirable features of a general framework.

### Structure and contributions

In this work, we develop two general methodologies that can represent Equations of State numerically, *exactly* satisfying conservation of energy and simultaneously incorporating phase transitions.

We view this problem within a geometric framework, where the symplectic character of thermodynamic manifolds (which, by definition, satisfy conservation of energy) is used to analyze our approach (“Problem statement” section). Based on this, we outline two EoS models that can generate thermodynamic data consistent with conservation of energy (“Methods” section): the additive (i.e., residual) correction (AGC) can do so *in the neighborhood of* phase transitions without changing their support, while the symplectic correction (SC) can transform regions where the phase transition *itself* occurs. We demonstrate applications of both methods through various computational examples and discuss their advantages and shortcomings. Using the output of these methods, we demonstrate that they can both be successfully utilized for further hydrodynamic computations (“Closing the loop: hydrocode computations” section), before concluding with several remarks (“Discussion” section). We use the Supplementary appendix to supply the reader with additional mathematical background and to expand on properties of the symplectic network architectures used.

Our novel contributions in this work are:

- We develop two neural EoS models (the AGC and SC) that are thermodynamically consistent *and* incorporate phase transitions. The SC works by smoothly (and symplectically) deforming an already-consistent model that may include phase transitions. This bypasses the complexity of incorporating discontinuities within network architectures, allowing us to make use of traditional smooth optimization algorithms.
- We provide a number of numerical examples using generated tabular SESAME data. We demonstrate that our predictions can be used to generate new tables on which hydrodynamic simulations can be performed.

## Problem statement

To enhance clarity, throughout this work we will use temperature (*T*) and volume (*V*) as independent variables, while treating entropy (*S*) and pressure (*P*) as dependent variables, obtained as the partial derivatives of the Helmholtz free energy (*A*). Reformulating for alternative choices independent and dependent variables is straightforward^36^.

### Setup

The EoS modelling problem can be appropriately framed in the context of symplectic geometry. Two relevant definitions are those of a symplectic manifold and Lagrangian submanifold:

#### Definition 2.1

A symplectic manifold $\mathcal {M}$ is an even-dimensional manifold equipped with a closed, non-degenerate differential two-form $\omega$, called the symplectic form. We often denote this pairing as $(\mathcal {M},\omega )$

#### Definition 2.2

A Lagrangian submanifold of a symplectic manifold $(\mathcal {M},\omega )$ with dimension 2*n*, is an *n*-dimensional submanifold $\Lambda \subset \mathcal {M}$ on which the symplectic form vanishes, i.e. $\omega \vert _\Lambda =0$.

We define thermodynamic phase space as $\Phi \simeq \mathbb {R}^4$ parametrized by (*T*, *V*, *S*, *P*). This space, equipped with the differential form $\omega =-dT\wedge dS-dV\wedge dP$ has symplectic structure. When viewing *S* and *P* as functions of the independent variables *T*, *V*, the condition that $\omega$ vanishes is equivalent to satisfying the following partial differential equation:

$$\begin{aligned} -\frac{\partial S}{\partial V}+\frac{\partial P}{\partial T}=0, \end{aligned}$$

which can be easily seen to be true by equality of mixed partials if there exists a $C^2$-scalar function *A*(*T*, *V*) such that

$$S(T,V)=-\frac{\partial A(T,V)}{\partial T},\quad P(T,V)=-\frac{\partial A(T,V)}{\partial V}.$$

Then, for any particular choice of thermodynamic potential *A*(*T*, *V*), the set of points that satisfy

1 $$\begin{aligned} \left\{ (T,V,S,P)\in \Phi :S=-\frac{\partial A}{\partial T},P=-\frac{\partial A}{\partial V}\right\} , \end{aligned}$$

will lie on a Lagrangian submanifold $\Lambda \subset \mathcal {M}$.

Thus, for a given chemical compound or element, its corresponding EoS is equivalent to a two-dimensional graph (and submanifold) $\Lambda$ in $\Phi$, which satisfies ‘conservation of energy’. We also refer to this property as ‘*energy-consistence*’. If an 2-dimensional submanifold is not generated by some scalar function *A*(*T*, *V*) then it does not satisfy the first law of thermodynamics.

We briefly expand on this formalism in Supplementary Appendix A.2, while a more rigorous recent account can be found in Ref.^37^.

For EoS that span multiple phases, it is known that *A*(*T*, *V*) is smooth within a single phase but may have kinks on the boundaries between phases. When phase transitions occur *A*(*T*, *V*) may only have well-defined first- or second-order derivatives but none of higher order. In that case, these regions respectively manifest as jumps or kinks in the corresponding Lagrangian submanifold $\Lambda$ (for details, see Supplementary Appendix A.3).

### Statement

Within this framework, we consider the following problem:

#### Problem 1

Given ‘input’ samples of an EoS, lying on a Lagrangian submanifold $\Lambda \subset \Phi$ and ‘target’ samples of a distinct EoS, lying on a different Lagrangian submanifold $\Lambda '\subset \Phi$, we are interested in learning, i.e., estimating, a correction map

2 $$\begin{aligned} f:\Lambda \rightarrow \Phi \,\,\,\textrm{such that}\,\,\, f(\Lambda )={\Lambda }'. \end{aligned}$$

Let our estimate correction map be $\hat{f}$. We require $\hat{f}$ to be energy-consistent, that is $\hat{\Lambda }'\doteq \hat{f}(\Lambda )$ must also be a Lagrangian submanifold of $\Phi$ that satisfies conservation of energy, and approximates $\Lambda '$

Satisfying conservation of energy is not inherently guaranteed by straightforward regression techniques between sampled submanifolds, but can still be achieved by imposing further structure on $\hat{f}$. However, energy-consistence is not overly restrictive, as it is satisfied by models that might be ‘very far from reality’. For example, modelling the energy as a constant with vanishing partial derivatives is thermodynamically consistent, but not realistic for any generic EoS.

From a physical point of view, $\Lambda$ can be thought of as a model we want to ‘fit’ to observations that are the samples of $\Lambda '$. We assume that the Lagrangian submanifolds are parametrized as

3 $$\begin{aligned} (T,V,S,P)\in \Lambda \subset \Phi ,\quad (T',V',S',P')\in \Lambda '\subset \Phi , \end{aligned}$$

and that there exist functions

4 $$\begin{aligned} q:(T,V)\mapsto Q,\quad q' : (T',V')\mapsto Q', \end{aligned}$$

that generate $\Lambda$, $\Lambda '$ respectively through Eq. (5)

5 $$\begin{aligned} \begin{aligned} S\doteq s(T,V)=-\frac{\partial A(T,V)}{\partial T},\\ P\doteq p(T,V)=-\frac{\partial A(T,V)}{\partial V}, \end{aligned} \end{aligned}$$

where $q,q'$ approximate the corresponding free energy functions $A,A'$ up to an additive constant. We specify the sense in which we expect $\hat{\Lambda }'\approx \Lambda '$ later on.

## Methods

We describe two qualitatively different approaches that attempt to solve the EoS approximation problem outlined in 1. Briefly, the *Graph* and *Additive Graph* corrections (GC, AGC) center around estimating and subsequently differentiating an ‘energy function’ *Q*, while the *Symplectic Correction* (SC) aims at manipulating a prescribed EoS in a manner that preserves conservation of energy.

### The (additive) graph correction

#### Formulation

A simple way obtaining a model that exactly conserves energy comes from exploiting the structure of Eq. (5) using the automatic differentiation capabilities of neural networks. We first observe that the (*T*, *V*) coordinates of $\Lambda$ can be identified with the coordinates $(T',V')$ of $\Lambda '$ since the Lagrangian submanifold can be seen as the 2-dimensional graph of a function with independent variables (*T*, *V*).

Then, in particular, if *q* is parameterized by a differentiable (fully-connected feed forward) neural network such that

$$\begin{aligned} q_{nn}:\mathbb {R}^2\rightarrow \mathbb {R},\quad (T,V)\mapsto \hat{Q}', \end{aligned}$$

obtaining its exact partial derivatives through automatic differentiation yields an energy-consistent model

6 $$\begin{aligned} \begin{aligned} \hat{f}_G:(T,V,S,P)&\mapsto (T,V,\hat{S}', \hat{P}')\\ \hat{S}'=-\frac{\partial q_{nn}}{\partial T}&,\quad \hat{P}'=-\frac{\partial q_{nn}}{\partial V}. \end{aligned} \end{aligned}$$

This method is effectively proposed in Ref.^20^.

Note that such a method does not require information about the dependent variables (*S*, *P*) on the input manifold $\Lambda$, which may be advantageous if these are not energy-consistent when given as input. However, in the case where we have consistent (full) samples of both an input and target submanifold, we may use the same formulation to produce a more ‘efficient’ *additive* correction as follows:

Under the assumption that we can decompose the energy functional of the target submanifold as

7 $$\begin{aligned} q'=q+q^+, \end{aligned}$$

where we only alter the energy functional of the input space *q* by an additive correction $q^+$ we have, by linearity of the partial derivative, that

8 $$\begin{aligned} S'=-\frac{\partial q'}{\partial T}=-\frac{\partial q}{\partial T}-\frac{\partial q^+}{\partial T}=S-\frac{\partial q^+}{\partial T},\quad P'=-\frac{\partial q'}{\partial V}=-\frac{\partial q}{\partial V}-\frac{\partial q^+}{\partial V}=P-\frac{\partial q^+}{\partial V}. \end{aligned}$$

Thus, estimating *only* this additive term with a neural network $q_\text {nn}^+\approx q^+$ allows us to formulate the following model

9 $$\begin{aligned} \begin{aligned} \hat{f}_G^+:(T,V,S,P)\mapsto&(T,V,\hat{S}', \hat{P}')\\ \hat{S}'= S - \frac{\partial q_\text {nn}^+}{\partial T},\quad \hat{P}'&=P-\frac{\partial q_\text {nn}^+}{\partial V}. \end{aligned} \end{aligned}$$

**Fig. 1:**
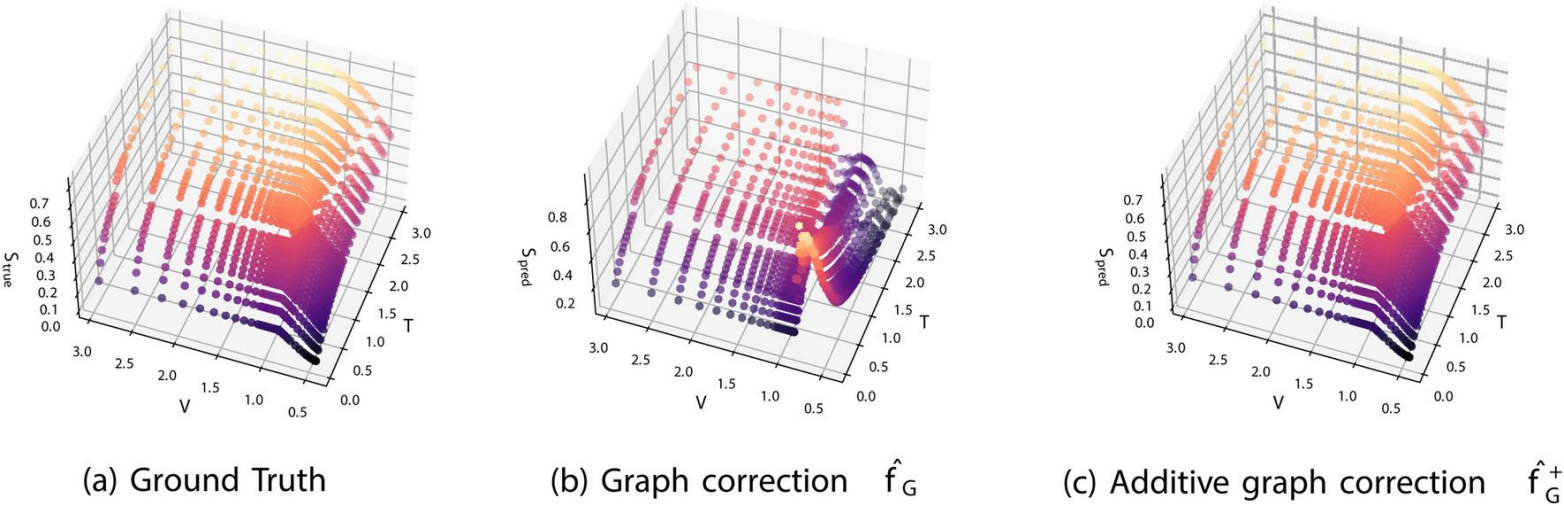
Ground truth entropy submanifold (*T*, *V*, *S*) compared to model predictions for the graph correction models $\hat{f}_G$ and $\hat{f}_G^+$ of “The (additive) graph correction” section. The color corresponds to the entropy value (*z*-axis).

This model makes full use of the ‘input’ manifold information and may generally require less training, i.e., may converge faster, if the input manifold $\Lambda$ is already a good approximation to $\Lambda '$. Furthermore, if $\hat{f}_G$ of the GC is parametrized by a smooth neural network, it will fail when attempting to estimate the energy functional *near* a phase transition, and the resulting dependent variables (*S*, *P*) may be severely distorted (as can be seen in the example of Fig. 1). In contrast, $f_G^+$ of the AGC uses pre-existing information about the energy functional of the input space implicitly, through the (*S*, *P*) values of the input submanifold. Thus, as long as *q* does not need to be ‘corrected’ exactly on the points where a phase transition occurs, this model is capable of producing faithful approximations of the target EoS submanifold. Note that both methods fail when the phase transition region itself needs to be corrected. We revisit the comparison between the methods in the “Discussion” section.

#### Computational example

##### Example 3.1

(Perturbed SESAME tables I) We demonstrate the behavior of the two methods (GC, AGC) near a phase transition by applying them to the following case study: We generate a multiphase EoS table for lead (Pb) as our ground truth target sample of $\Lambda '$, and artificially perturb it near a phase transition region, to produce a second, perturbed table, as our input samples of $\Lambda$ (for details on the data sets and architectures used, see Supplementary Appendix C).

We train two models $\hat{f}_G$ and $\hat{f}_G^+$ to estimate the point-wise correction between the two manifolds. For both, we minimize the supervised objective

10 $$\begin{aligned} \mathcal {L}=\frac{1}{N}\sum _{i}^N\left( \left\| (\hat{S}'_i,\hat{P}'_i)-(S'_i,P'_i)\right\| _2^2\right) , \end{aligned}$$

to achieve comparable training losses (on the order of $10^{-2}$). However, upon plotting the predicted entropy variables (Fig. 1) we observe that $\hat{f}_G$ has a qualitatively wrong feature, a large jump, stemming from the inability of the smooth model to capture phase transitions. This is not observed in the case of the additive correction. Fundamentally, GC fails due to the smoothness of the approximating networks (fully connected with $\tanh$ activation functions), which cannot appropriately approximate the underlying free energy. While the AGC is smooth as well, it ‘retains’ the phase structure of the input submanifold and thus is capable of approximating the target EoS.

### The symplectic correction

#### Formulation

An alternative approach to the correction problem that still guarantees energy conservation makes use of symplectic diffeomorphisms, or ‘symplectomorphisms’, of the ambient thermodynamic space $\Phi$. Informally, these are a class structure-preserving diffeomorphisms between symplectic manifolds that preserve conservation laws of their input space. For details, refer to Supplementary Appendix A.

Universal parametrizations of symplectomorphisms via neural networks include the SympNet ^38^ and HenonNet ^39^, which are based on a characterization developed in Ref.^40^. In our work, we specifically modify the implementation proposed in Ref.^39^ such that it is *L*-bi-*Lipschitz*. Universality guarantees that a large enough architecture will be expressive enough to approximate the desired correction map, while Lipschitz control offers some stability during training with larger learning rates. We expand on the architecture and its properties in Supplementary Appendix B.

If $\varphi :\Phi \rightarrow \Phi$ is a symplectomorphism, then the image $\varphi (\Lambda )$ of any Lagrangian submanifold is *also* Lagrangian. This motivates us to introduce a symplectic neural network $\varphi _\text {nn}:\Phi \rightarrow \Phi$ whose weights are chosen to ensure $\varphi _\text {nn}(\Lambda )\approx \Lambda '$.

The desired correction map $\hat{f}_s$ is given by the restriction of $\varphi _\text {nn}$ to the input submanifold $\Lambda$.

In this approach, we may choose to identify the independent variables ((*T*, *V*) with $(T',V')$) of the input and target submanifolds, similarly to “The (additive) graph correction” section. However, under this identification of the independent variables, phase transitions are ‘locked in place’. This means that the approximation map will attempt to smooth out kinks at some location and create kinks in another if they do not line up between $\Lambda$ and $\Lambda '$ a priori. (For an illustrative example, see Example B.1 in the Supplementary Appendix).

Instead, we may choose to consider more general *manifold* losses between $\Lambda$ and $\Lambda '$ where no pointwise correspondence is specified. These are metrics that consider the manifold as a whole object (e.g., Hausdorff distance between two Euclidean-embedded submanifolds, sampled as point clouds). We expand on the types of losses considered here in Supplementary Appendix B. Importantly, considering manifold distances does not fix the location of the phase transitions, only their *type*:

Since $\phi _\text {nn}$ is a diffeomorphism, it must preserve the regularity of its pre-image, and so if $\Lambda$ has a phase transition, $\Lambda '$ will also have *the same type* of phase transition. This is desirable, since it guarantees that we have not altered the qualitative structure of the element we are studying when estimating the correction map. Thus, by using symplectomorphisms on ambient space, we guarantee that the correction is energy consistent *and* gain additional freedom in our ability to *move* and *model* phase transitions.

Importantly, this also gives rise to the idea of ‘templating’, where a general template (that is qualitatively correct in some sense) is prescribed and molded, i.e., fitted,to a particular data set, not necessarily using ML methods. This framework is far more general than its use in the specific work (Supplementary Appendix A.4).

**Fig. 2:**
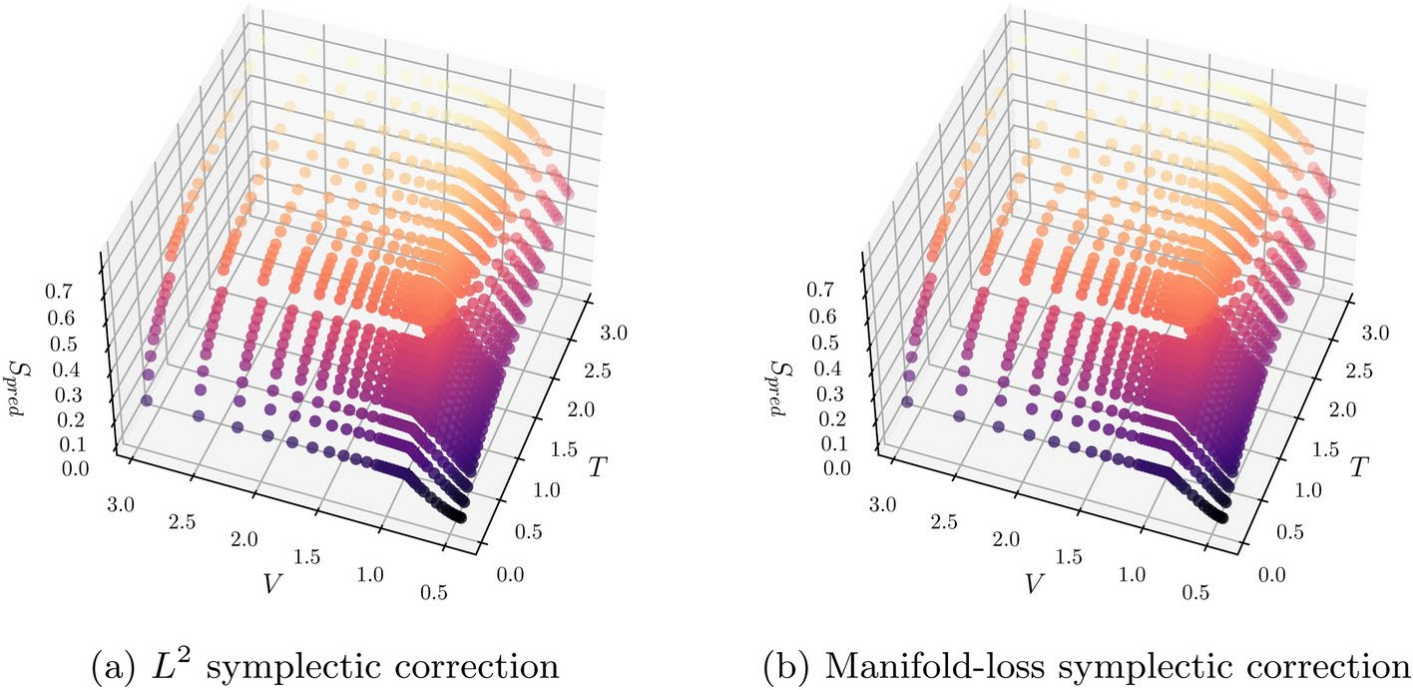
Entropy submanifold (*T*, *V*, *S*) for a symplectic network “The symplectic correction” section optimized under $L^2$ (left) and a permutation invariant Hausdorff-like loss (right). The color corresponds to the entropy value (*z*-axis).

#### Computational examples

##### Example 3.2

(Perturbed SESAME tables II) In the identical setting of Example 3.1, we estimate correction maps $\hat{f}_s$ by minimizing both the $L_2$ (pointwise) and manifold losses. These are given by Eqs. (14) and (16) in Supplementary Appendix B.3 respectively. In both cases, depicted in Fig. 2, the discrepancy between the estimate and target submanifolds is small after successful training (see Supplementary Appendix, Table 3).

We include this example to demonstrate that the symplectic correction also overcomes the phase transition issue demonstrated in Example 3.1, and can be optimized through the two different types of objectives that we consider. However, a small residual and consistent plots may be insufficient to assess the quality of a fit. We consider additional validation steps through hydrodynamic simulations in the “Closing the loop: hydrocode computations” section.

**Fig. 3:**
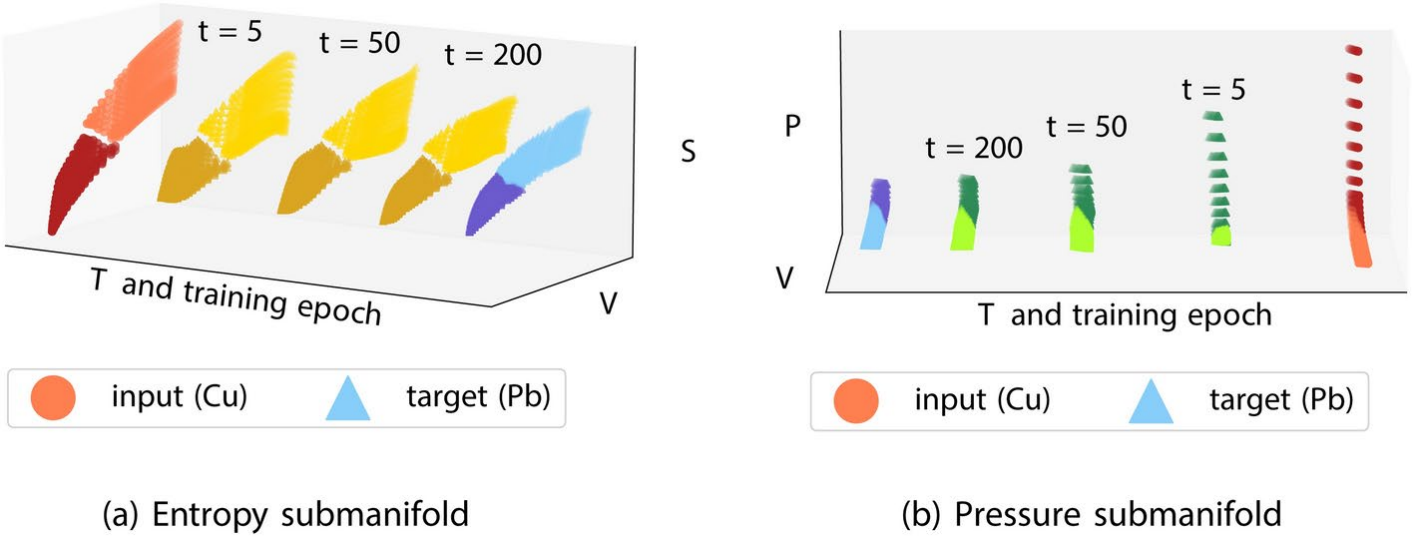
Training trajectory for the entropy (left) and pressure (right) submanifolds as a symplectic network learns the map from Cu (red) to Pb (blue). The *x*-axis is translated at each training time to produce the figures: in reality the (*T*, *V*) support of the submanifolds is identical, with the differences observed on the dependent variables.

##### Example 3.3

(Mapping Cu to Pb) As in example Example 3.1, we generate SESAME tables for lead and copper (Cu). We consider the part of the table supported on a grid of (*T*, *V*) in the same range for both elements, which also includes a solid-to-liquid phase transition. We then estimate a correction $\hat{f}_s$ to map one table onto the other, noting that the dependent variables (*S*, *P*) cover different ranges of values for each element, as presented in Fig. 3.

While this example may seem extreme at first glance, it primarily serves to show that the symplectic correction can be implemented between manifolds that have a ‘large’ distance between them, i.e., they are not within a small perturbation of each other, in example Example 3.1.

We note that, if the input sesame table is thermodynamically inconsistent (which is generically the case due to interpolation errors) the output of the symplectic network will *also* be inconsistent. This does not prohibit us from solving the corresponding optimization problem.

##### Example 3.4

(Analytic correction)

Finally, we construct a simple, *exact* EoS using the Ideal Gas and Sackur–Tetrode^41,42^ equations. This yields the following expression for the Helmholtz free energy:

11 $$\begin{aligned} A(T,V)\doteq \frac{3 k_B T}{2}-k_B T \left( \ln (2 \sqrt{2} \pi ^{3/2} V \left( \frac{k_B m T}{h^2}\right) ^{3/2})+\frac{5}{2}\right) , \end{aligned}$$

where $k_B$ is the Boltzmann constant, *h* is the Planck Constant, and *m* is the mass of a single particle. To demonstrate how this may be used as an editable template, we consider a smooth, piece-wise addition which acts as a first-order phase transition for the modelled material (Eq. (12)). (In a similar manner, one may add an arbitrary number of phase transitions of different types and at different initial locations). We consider the initial location (*l*) and scale (*s*) of the phase transition as two positive, real-valued parameters which can later be changed:

12 $$\begin{aligned} A_{(l,s)}(T,V)= {\left\{ \begin{array}{ll} \frac{3 k_B T}{2}-k_B T \left( \ln (2 \sqrt{2} \pi ^{3/2} V \left( \frac{k_B m T}{h^2}\right) ^{3/2})+\frac{5}{2}\right) ,& V<l\\ \frac{3 k_B T}{2}-k_B T \left( \ln (2 \sqrt{2} \pi ^{3/2} V \left( \frac{k_B m T}{h^2}\right) ^{3/2})+\frac{5}{2}\right) -s(V-l),& V\ge l\\ \end{array}\right. }. \end{aligned}$$

This free energy will produce a phase transition along $V=l$ of magnitude *s*, which will result in a jump for pressure at the corresponding location.

Using this equation, we generate a sample of the analytic thermodynamic submanifold using the same (*T*, *V*) values that appear in a region of the Cu SESAME table. We then proceed to estimate the correction between the analytic model and the tabular data using a symplectic network.

**Fig. 4:**
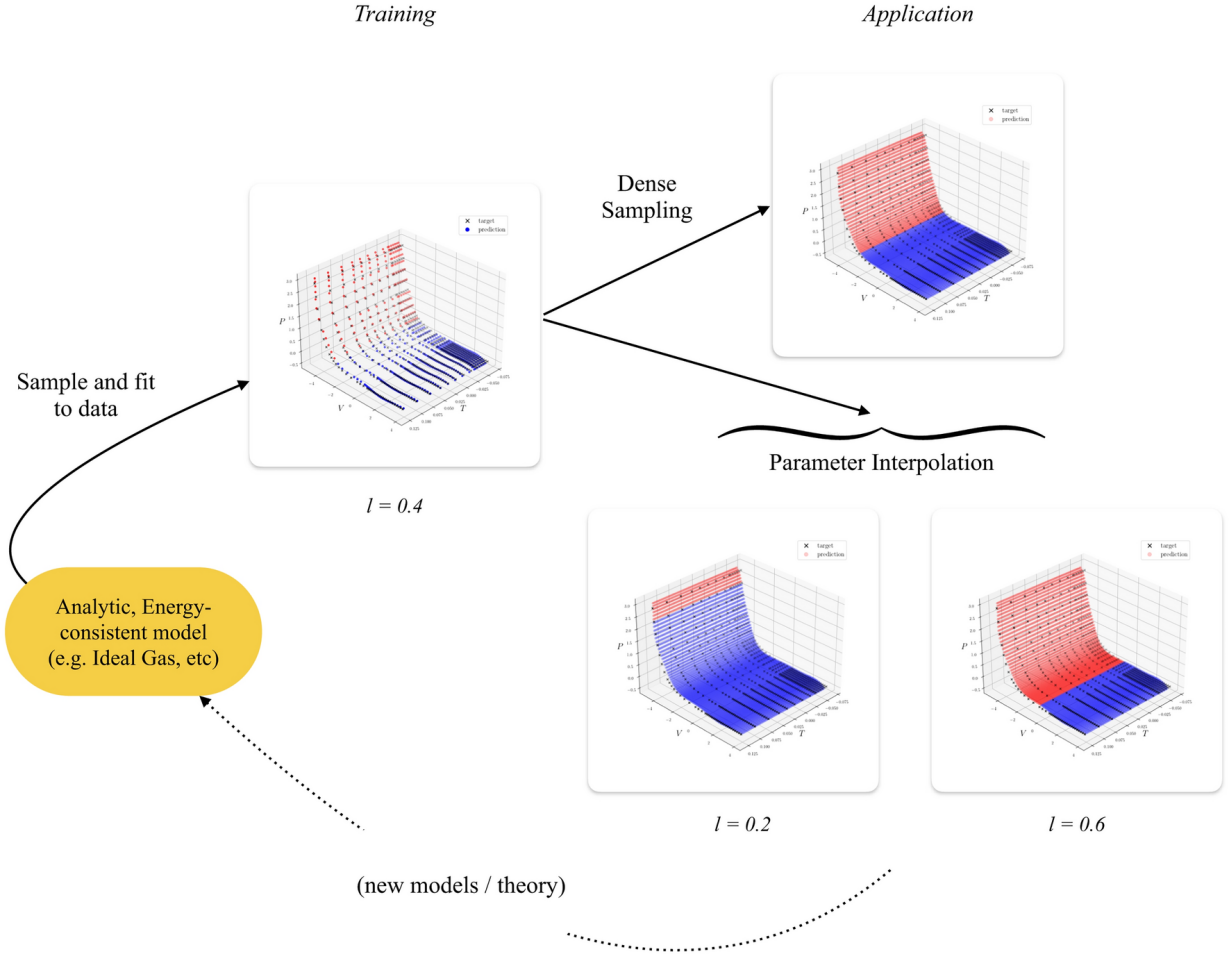
Representation of the workflow using an analytic template (Eq. (12)) to fit a region of SESAME table data (Cu) using a symplectic network. After fitting, we can sample arbitrary points on the fitted submanifold, thus producing dense samples. The parameters can also be modified to better fit data, (in this case different values of *l* are shown, representing the location of the phase transition).

After convergence, we can use the analytic model to (a) generate denser samples of the tables, and (b) a posteriori modify parameters of the input template to sample an EoS in the region of the target, in an interpretable manner. Both of these capabilities can be broadly exploited in downstream tasks (Fig. 4). Note that, assuming the network architecture used is differentiable, the output can also be differentiated with respect to parameters, thus making the method amenable to (a posteriori) parameter inference using gradient descent methods^43^.

This example demonstrates that a relatively ‘simple’ building block EoS, such as the Ideal Gas model, can be modified to construct template EoSs with arbitrarily complicated phase diagrams. These can be fit to data (using the proposed methods). The main difficulty with such an approach lies in parametrizing the phase boundaries, which may have complex geometric features.

## Closing the loop: hydrocode computations

**Fig. 5:**
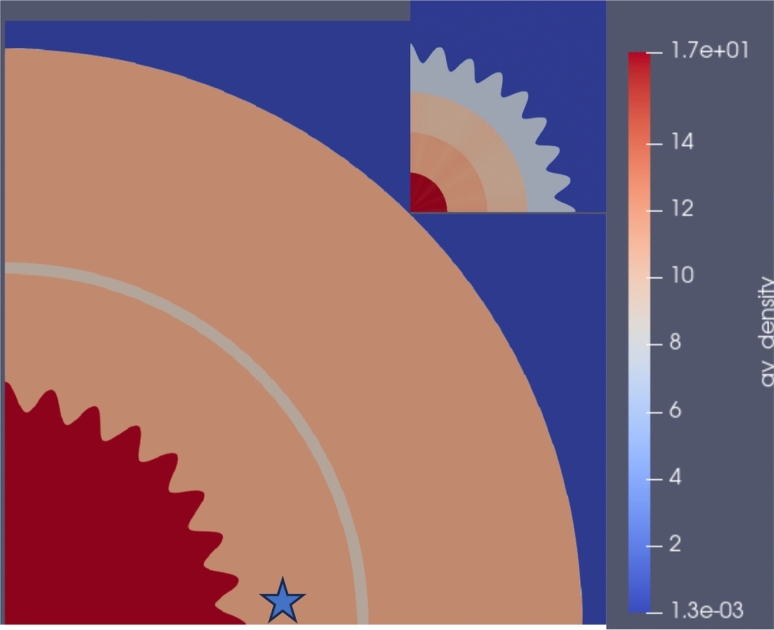
Initial system Pb–Cu system with tantalum driving shells and location of Lagrangian tracer.

EoS models have numerous applications, one of which includes hydrodynamic simulations that track trajectories in thermodynamic phase space under specified geometric, boundary, and initial conditions. In this context, we explore the efficacy of our ML-generated EoS tables in facilitating hydrodynamic simulations where the thermodynamic trajectory evolves though multiple phase transitions. Our focus extends to simulations where hydrodynamic responses are intricately tied to the properties of the EoS. To this end, we delve into a specific hydrodynamic scenario characterized by the emergence of a Richtmyer–Meshkov instability (RMI), triggered by the interaction of a shock wave with a non-uniform interface between two materials of differing properties, such as density variations. An illustrative example of this phenomenon is depicted in Fig. 5, showcasing an explosive scenario where a high-density material, Tantalum (Ta), encapsulated within four concentric shells each imparted with an initial outward velocity, propels into a composite medium of lead and copper.

**Fig. 6:**
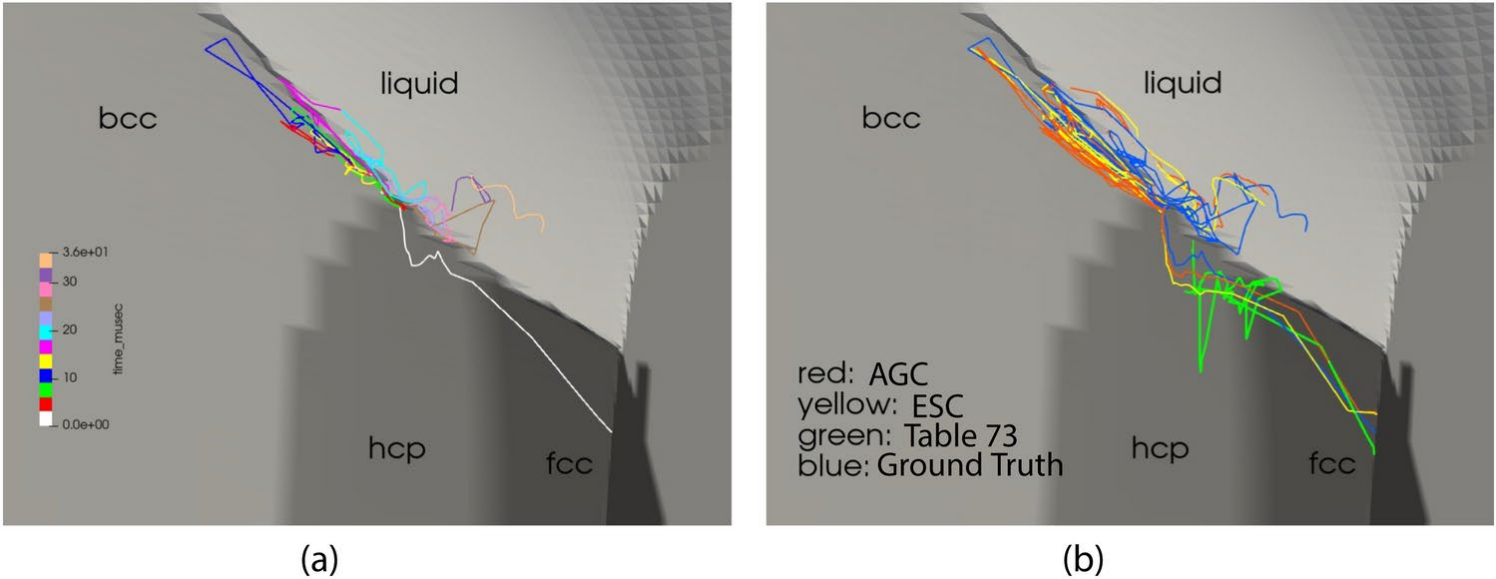
**(a)**, Thermodynamic trajectory of Lagrangian tracer in Lead. **(b)** Comparisons of the thermodynamic trajectory of the Lagrangian tracer using the baseline, initially perturbed Pb EoS table, and the reconstructed EoS tables.

Leveraging the geometric configuration illustrated in Fig. 5 and considering the initial outward velocities of the Ta shells (detailed in Supplementary Appendix D), we employed the baseline Pb SESAME Table 3206 (ground truth) to generate a representative thermodynamic trajectory. This trajectory pertains to a Lagrangian tracer situated within the Pb material, as presented in Fig. 6a.

**Fig. 7:**
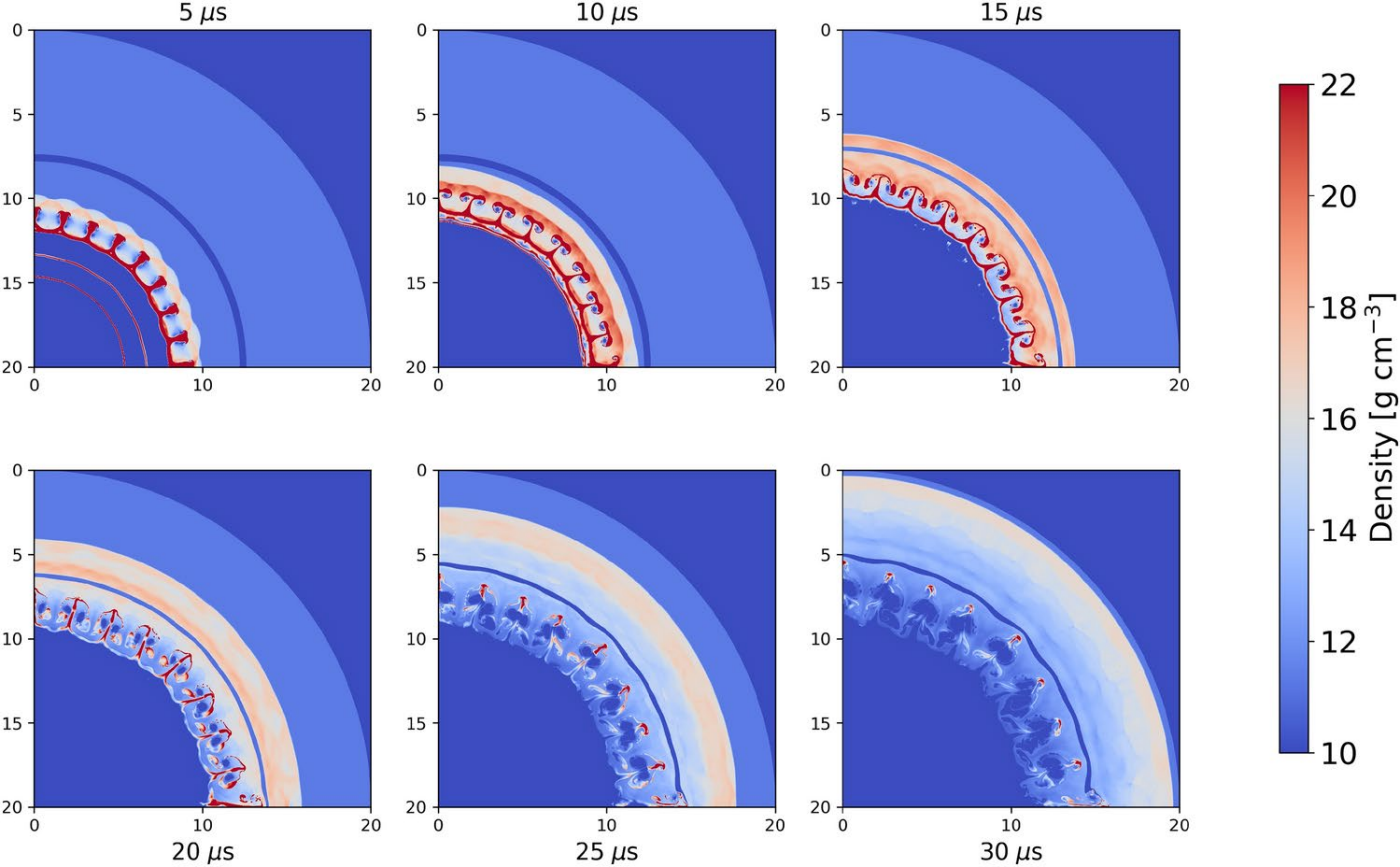
Density fields for Pb Sesame Table 3206 g/cm$^3$.

As may be observed from examination of Fig. 6a, the hydrodynamic trajectory of the tracer passes through four EoS phases during the course of the simulation. Representative density fields for the simulation using the baseline EoS Sesame Table 3206 for Pb are presented in Fig. 7.

Examination of Fig. 7 indicates the development of a RMI as the shocks generated via the initial outward moving shock interact with the irregular interface at the Ta/Pb interface. Additional complexity in the instability development is achieved by the subsequent interaction of the reflected shocks at the Pb–Cu interface.

**Fig. 8:**
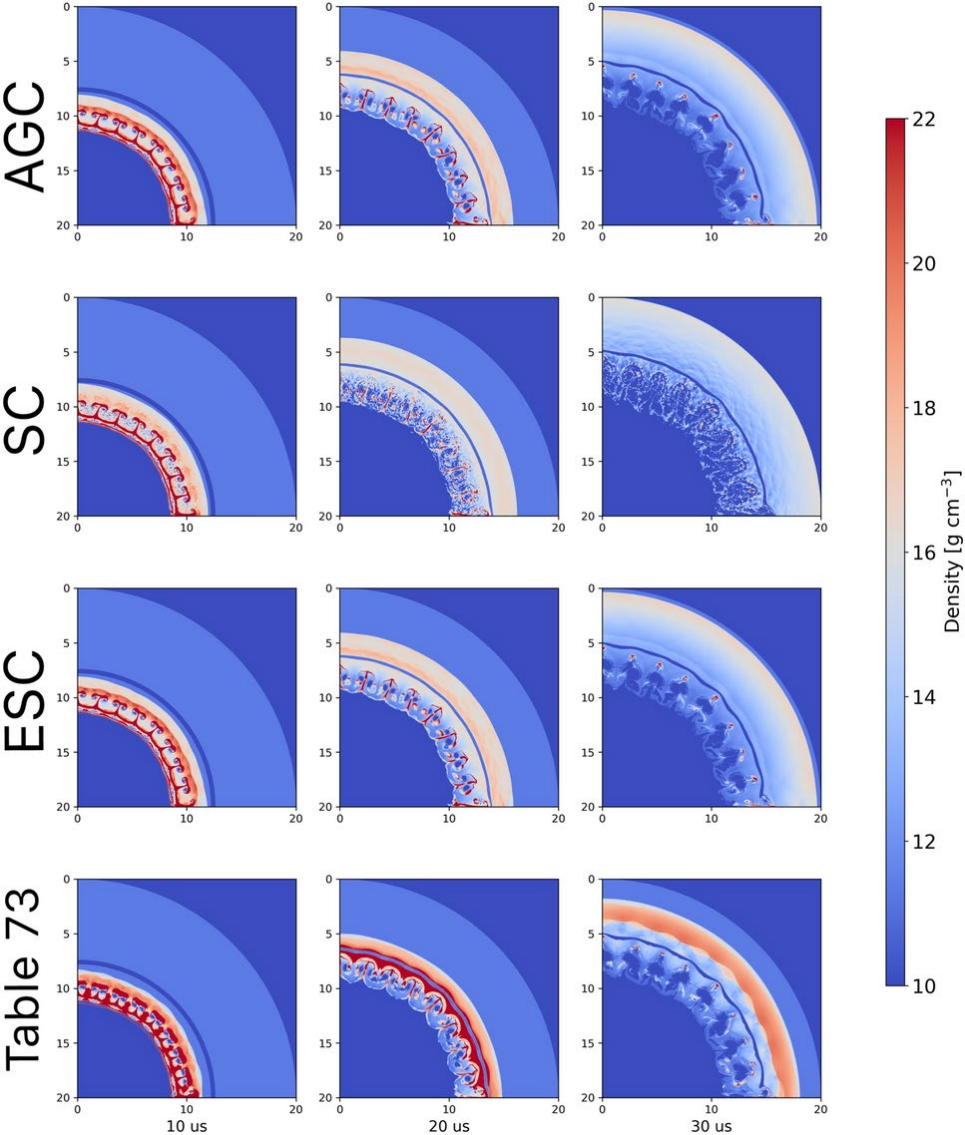
Reconstructed density fields for AGC (first row) and SC (second row) and ESC (third row) Networks along with density field obtained with starting perturbed Pb EoS Table 73 (fourth row).

To examine the impact of the reconstructed EoS on the hydrodynamic simulation, we consider a similar setting to that of Example 3.1. The ground truth table for Pb (Sesame 3206) is used to compute a set of trajectories for a set of markers $\mathfrak {M}=\left\{ m_i\right\} _{i=1}^M$ in thermodynamic space:

13 $$\begin{aligned} \mathcal {T}=\left\{ \mathcal {T}_t^{m_i}:\mathcal {T}_t^{m_i}=(T_t^i,V_t^i,S_t^i,P_t^i)\in \Phi , m_i\in \mathfrak {M}, t\in [T_\text {max}]\right\} , \end{aligned}$$

for discrete time steps indexed by *t* that have length $T_\text {max}$. We then map the perturbed Pb Sesame Table (Table 73, see Supplementary Appendix D for details) to the ground truth table, using both the AGC and SC corrections, and compare the corresponding hydrodynamic trajectories and density fields to those corresponding to the ground truth table. Figure 8 presents density fields using the AGC, SC, and the early, mid, and late times.

**Fig. 9:**
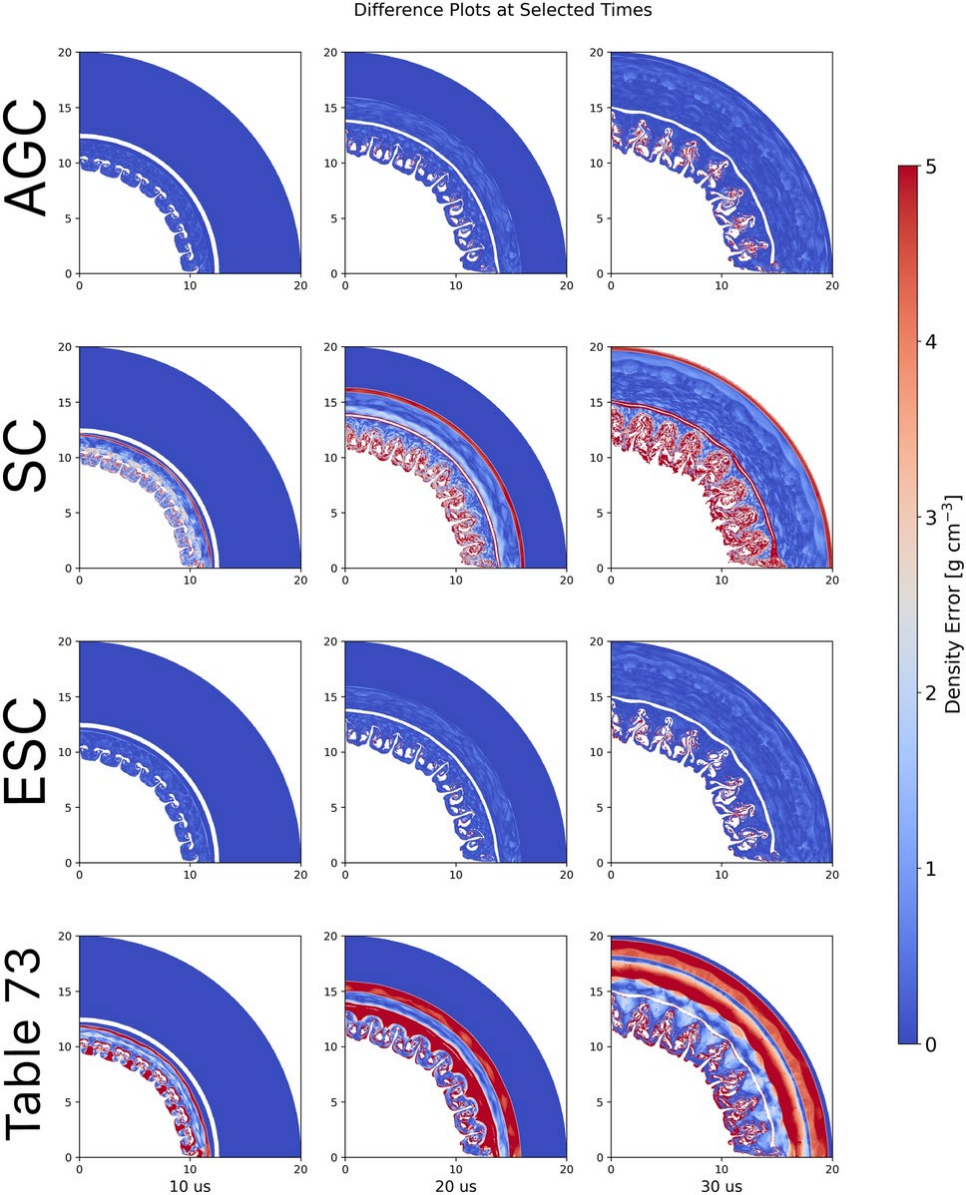
Residuals between the Reconstructed Pb Tables and the Perturbed Pb table with the baseline Pb EoS Table 3206.

We note that, to perform these simulations we require a completed SESAME table, for which it is necessary to have an estimate of the free energy in addition to (*T*, *V*, *P*, *S*). This is readily available for AGC but not for the symplectic correction. To remedy this issue, we extend the symplectic network in Supplementary Appendix B.2 to compute the induced effect of the SC on the free energy variable. This transformation resembles a Legendre map, and we refer to it as the extended-symplectic correction (ESC). Using this formulation, the free energy can be either completed after fitting the original SC, or fitted along with the other target variables during training.

Several observations are apparent in examining Fig. 8.

1. There are significant differences between the hydrodynamic simulations using the initial Pb EoS perturbed table and those obtained using the Sesame 3206 table
2. Both the AGC and SC networks are able to produce excellent reconstructed Sesame Tables. Furthermore, when these reconstructed tables are utilized within the frame work of a hydrodynamic simulation excellent matches to both the EoS tables as well as density fields are achieved.

**Fig. 10:**
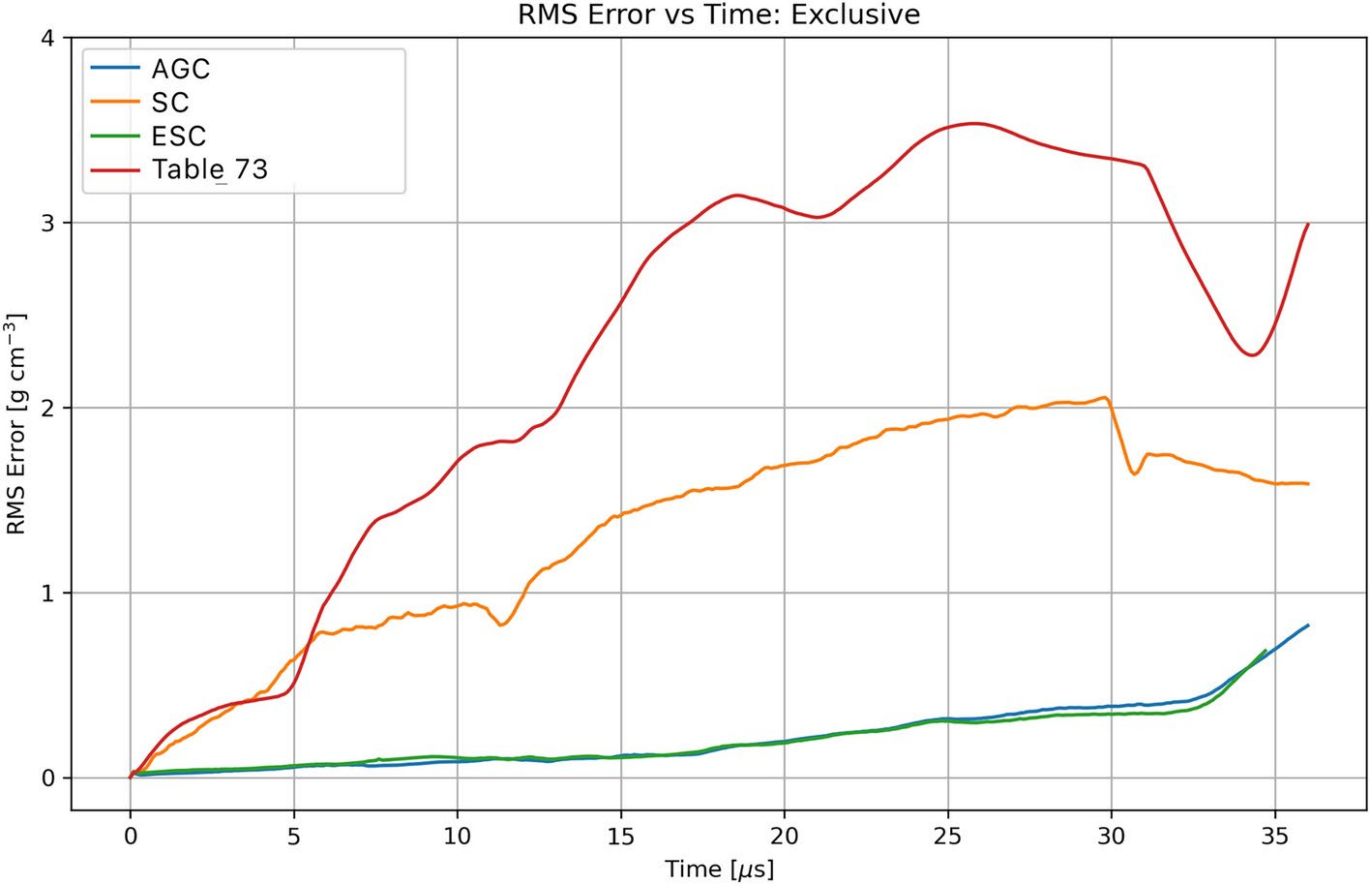
Root-mean squared differences between reconstructed and perturbed EoS Tables with baseline Pb EoS Table 3206.

Quantitative comparisons of the density differences between the respective tables and SESAME Table 3206, along with those using the perturbed Sesame Table, are provided in Figs. 9 and 10.

Finally, comparisons of the respective hydrodynamic paths using the Lagrangian tracer embedded within the Lead are provided in Fig. 6a. As indicated in Fig. 6b, the tracers using the respective networks, closely follow the path of the baseline table. However, the tracers using the initial perturbed EoS table indicate a somewhat different trajectory within the hcp phase.

## Discussion

Throughout this work, we propose two computational approaches to representing EoS with neural architectures. Both are capable of incorporating phase transitions in their models, significantly enhancing the applicability of such neural models to applications that may require multiphase EoS. In the case of symplectic networks, this is achieved through *templating*.

Combining the computational capabilities of ML algorithms, in particular neural networks, with EoS allows us to build black- or grey- boxes that fit experimental observations or consistently (in the sense of the first law of thermodynamics) interpolate between observations and theoretical limits. This may be a particular advantage for materials and phase-space regions where performing experiments may be expensive or time-consuming, thus making it difficult to obtain a dense sample of the true EoS.

Our problem approach, that of a ‘manifold correction’, is a particular choice that easily generalizes to other settings. As future work, we propose extensions in (a) the direction of generative EoS modeling (where an appropriate physical model is not available), and (b) the issue of designing proper, general templates that can be used for multiple compounds or elements. Finally, the symplectic correction, which is more flexible in the sense that it can ‘modify’ phase transition regions, is limited since it cannot be applied in a stable manner for multiscale EoS data sets. This is also an important issue to be addressed. Using manifold-type losses to optimize allows movement of phase transition regions. In this work we do this in an unsupervised manner, in which we allow the network to appropriately move the discontinuity set to minimize the corresponding objective. A supervised approach in the future may be more robust computationally, in which phase boundaries between the input and target EoS are identified prior to optimization and subsequently matched. Furthermore, the manifold-type losses considered in this work are computationally expensive, since distances are computed between large sets of points. Considering approximations of Wasserstein distances between EoS manifolds (using Sinkhorn-type algorithms^44^) may allow for faster and more robust optimization of the symplectic network architecture.

## Supplementary Information

Supplementary Information.

## Data Availability

The accompanying code will be available after publication upon reasonable request to the authors. Data related to SESAME tables can be requested through Los Alamos National Laboratory at https://www.lanl.gov/org/ddste/aldsc/theoretical/physics-chemistry-materials/sesame-database.php.
