# Supplementary material for: Neural network representations of multiphase Equations of State
Source: Sci Rep. 2024 Dec 5;14:30288. doi: 10.1038/s41598-024-81445-4 (PMC11618593; doi:10.1038/s41598-024-81445-4)
Supplement: Supplementary file 1 — Supplementary Information. [file 41598_2024_81445_MOESM1_ESM.pdf]

Supplemental Material for
Neural Network Representations of Multiphase Equations of State

George A. Kevrekidis[1,2,*], Daniel A. Serino[1], M. Alexander R. Kaltenborn[1], J. Tinka Gammel[1], Joshua W. Burby[1,2], and Marc L. Klasky[1,*]

[1] *Los Alamos National Laboratory, Los Alamos, NM, USA*
[2] *Department of Applied Mathematics and Statistics, Johns Hopkins University, Baltimore, MD, USA*
[3] *Department of Physics, University of Texas at Austin, Austin, TX, USA*
[*] *Corresponding Author*

June 28, 2024
LA-UR-24-25494

A Differential Geometry

A.1 Theory

Definition A.1 (Symplectic Manifold)**.** A manifold $\mathcal{M}$ is called symplectic if it has even dimension, and is equipped with a closed non-degenerate differential two-form ω. We denote this as $(\mathcal{M}, \omega)$.

Example A.1. The usual Euclidean space $\mathbb{R}^{2n}$ parametrized by $(x^1, ..., x^n, y^1, ..., y^n)$ is symplectic when equipped with the two-form

$$\omega = \sum_{i=1}^{n} dx^i \wedge dy^i$$

Definition A.2 (Lagrangian Submanifold)**.** Given a symplectic manifold $(\mathcal{M}, \omega)$ of dimension $2n$, a submanifold Λ is called Lagrangian if it has dimension n and the restriction of the symplectic form on it vanishes $(\omega|_\Lambda = 0)$.

In this work, thermodynamic phase space Φ is treated as a symplectic manifold ($\simeq \mathbb{R}^4$) and the thermodynamic data for any given element or compound lie on a Lagrangian submanifold Λ of Φ, a consequence of satisfying conservation of energy. We prove this result in the following section.

Definition A.3 (Symplectomorphism)**.** A symplectomorphism, or symplectic diffeomorphism ϕ is a map between two symplectic manifolds $(\mathcal{M}, \omega)$ and $(\mathcal{M}', \omega')$ that 'preserves' the symplectic form, in the sense that the pullback satisfies

$$\phi^* \omega' = \omega$$

Symplectomoprhisms are diffeomorphisms (smoothly invertible) and volume-preserving maps. In our work, we identify ω with ω' since we consider automorphisms of the thermodynamic phase space Φ.

A.2 The Geometric Structure of EoS Manifolds

A **Thermodynamic Variable** is a physical quantity associated with the state of an atomic substance or chemical compound. Examples include Temperature (T), Pressure (P), Volume (V), Entropy (S), Internal Energy (U), Helmholtz Free Energy (A), Enthalpy (H), etc. Each variable is real-valued, and possibly non-negative.

For a given element or compound, these quantities are related, and in general specify a two-dimensional submanifold of Euclidean space. Importanty, such thermodynamic submanifolds are generically **graphs** of a function, i.e., knowing two of these quantities allows one to infer the rest uniquely. An **Equation of State** (EoS) is a mathematical model that describes the relationship between these variables.

In general, we may split the variables into three types:

1. Independent Variables (e.g., two of $\{T, V, P, S, \text{etc.}\}$).
2. Thermodynamic potential functions, (e.g., $\{U, H, A, \text{etc.}\}$), seen as scalar functions of the independent variables, uniquely determined by them for a given element.
3. Dependent variables (e.g., the remainder of $\{T, V, P, S, \text{etc.}\}$) which are functions (in particular derivatives) of the energy functions.

We first establish the relationship between EoS and Symplectic Geometry. Henceforth we use (T, V, P, S, A) as our thermodynamic variables of choice, and we will in particular treat (T, V) as the independent variables, A as the corresponding energy functional, and

$$\begin{aligned} S &\doteq s(T,V) = -\frac{\partial A(T,V)}{\partial T} \\ P &\doteq p(T,V) = -\frac{\partial A(T,V)}{\partial V} \end{aligned} \tag{1}$$

as the dependent variables. This is done to demonstrate both theory and applications with some consistence; however, one may reformulate the statements with any other suitable choice of variables. Indeed, a choice of thermodynamic potential usually implies a particular choice of independent variables [1].

Definition A.4 (Thermodynamic Phase Space)**.** Thermodynamic Phase Space Φ is a 4-dimensional symplectic manifold when parametrized by

$$\Phi = (T, V, S, P) \in \mathbb{R}^4 \tag{2}$$

and equipped with the canonical symplectic two-form

$$\omega = dT \wedge dS + dV \wedge dP \tag{3}$$

In this space, the **First Law of Thermodynamics** can be written in differential form as

Definition A.5 (Conservation of Energy)**.** Given the symplectic manifold Φ, the first law of thermodynamics can be expressed as the differential one-form

$$dA = -SdT - PdV \tag{4}$$

Furthermore, for a given element or compound, its thermodynamic data lie on a two-dimensional **Lagrangian submanifold** $\Lambda \subset \Phi$ which satisfies conservation of energy, a consequence of satisfying the first law. We use the term '**energy-consistent**' to describe submanifolds and models that satisfy this property.

Additionally, we observe that:

$$\begin{aligned} 0 = d(dA) &= -\frac{\partial S}{\partial V} dV \wedge dT + \frac{\partial P}{\partial T} dV \wedge dT \\ &= \left(\frac{\partial P}{\partial T} - \frac{\partial S}{\partial V}\right) dV \wedge dT \end{aligned}$$

which yields the equivalent constraint to ω vanishing in the form of a differential equation.

A.3 Phase Transitions

This thermodynamic submanifold Λ is assumed to be smooth within a single phase of the compound or material (i.e., the graph of a C^∞ function). However, it is not smooth across phase boundaries, locations in phase space where phase transitions occur, where Λ represents a material with multiple phases. Classically, according to the **Ehrenfest classification** [2], phase transitions are classified as

1. 1st Order, when the first derivative of the energy function (A) is discontinuous.
2. 2nd Order, when the first derivative of the energy function (A) is well-defined, but its second order derivative is discontinuous.

In the latter case, Λ is smooth almost everywhere, and is the graph of a Lipschitz function. Phase transitions appear as 'kinks' or 'ridges' in projections of the embedded submanifold Λ, and we also refer to them simply as 'discontinuities', due to the ill-defined tangent space at the particular points.

Phase transitions are 'interesting' but irregular regions of phase space that must be modelled carefully to obtain an accurate and energy-consistent multiphase EoS.

A.4 Templating

Our proposed approach (Section 2) can be seen through the lens of Templating: Starting from a qualitatively correct, exact model, learning a transformation that maps the model to observed data. In the EoS case, the qualitative feature that must be present in the template is primarily the type (and topology) of phase transitions. However, designing templates is not a trivial task, especially if one expects to find global (multiscale, multiphse) EoS models that accommodate several elements. The main advantage of templating, however, is that one may use continuous techniques to manipulate 'discontinuous' templates, effectively removing the need to incorporate the discontinuities into new architectures. They further allow us to use the expressivity of neural networks while retaining some control over interpretable features, as in Example 3.4. Additionally, such parametric dependence grants a generative capability, in which parameters can be further *fitted or sampled* after initial training ([3]). For a general reference on 'deformable templates', see [4].

B Symplectic Networks

B.1 Formulation

Symplectic neural networks are parametric families of functions f_θ that exactly preserve the symplectic structure of their input (a symplectic manifold) under any choice of parameters θ. In particular, if $\mathcal{M}$ is a symplectic manifold, then so is $\mathcal{N} \doteq f_\theta(\mathcal{M})$.

In this work we use the *HénonNet* architecture introduced in [5]. We restate the following definitions for completeness.

Definition B.1 (Hénon Map)**.** Given a smooth function $V : \mathbb{R}^n \to \mathbb{R}$ and a constant vector $\eta \in \mathbb{R}^n$, a Hénon map is defined as

$$H[V, \eta] : \begin{pmatrix} x \\ \\ y \end{pmatrix} \mapsto \begin{pmatrix} y + \eta \\ \\ \boldsymbol{\nabla} V(y) - x \end{pmatrix} \tag{5}$$

for $x, y \in \mathbb{R}^n$.

It is easy to check that both this map and finite compositions of the form $H[V_1, \eta_1] \circ H[V_2, \eta_2] \circ ... \circ H[V_n, \eta_n]$ are symplectic, for arbitrary choises of V_i and η_i.

The underlying result that makes maps of this form particularly useful is that for appropriate V and η, they are dense within the family of symplectic maps on $\mathbb{R}^{2n}$. This is formally captured in [6].

In practice, V is the only part of the structure that is estimated by a (deep) feed-forward neural network (FNN). Since FNNs are universal approximators of differentiable scalar functions [7, 8] $f : \mathbb{R}^n \to \mathbb{R}$, the Hénon Net architecture is capable of approximating any symplectic map.

Definition B.2 (Hénon Layer)**.** Given a Hénon map $H[V, \eta]$, the corresponding Hénon Layer $L[V, \eta]$ consists of the four-fold composition of the original map:

$$L[V, \eta] = H^4[V, \eta] \tag{6}$$

Note that this is not a necessary step to design a network-based symplectic map approximator. Nevertheless, the choice of using the layer as a basis block is stable in practice. Observe that for $V \equiv 0$, the $L[0, \eta]$ is the identity map in $\mathbb{R}^{2n}$.

Definition B.3 (Hénon Network)**.** A Hénon network H_{nn} of K layers is a map formed by composing K distinct Hénon layers $\mathbf{L} = \{L[V_i, \eta_i]\}_{i=1}^{K}$:

$$H_{\text{nn}}[\mathbf{L}] = L_K \circ ... \circ L_1 \tag{7}$$

where $L_i = L[V_i, \eta_i]$.

We also observe that Hénon maps have a closed-form inverse [9, Remark 2.2]:

Definition B.4 (Hénon Map Inverse)**.** Given a Hénon map $H[V, \eta]$ it's inverse is the map defined as

$$H^{-1}[V, \eta] : \begin{pmatrix} x \\ \\ y \end{pmatrix} \mapsto \begin{pmatrix} \boldsymbol{\nabla} V(x - \eta) - y \\ \\ x - \eta \end{pmatrix} \tag{8}$$

Thus, composing these such inverses in an appropriate manner gives a closed form inverse for any particular instance of a Hénon network architecture. This is convenient computationally since an inverse network is readily available when needed, but also allows us to initialize the network architecture to be the identity map before training (by composing with its original inverse which is not updated). This is useful when the the input manifold is already a good approximation of the target manifold (such as in the EoS case discussed where Λ is originally estimated as an approximation to Λ').

B.2 Contact Extension

After training the symplectic networks, it may be necessary to lift back to an integral variable (i.e., the energy potential in the case of EoS). This computation is needed, for example, when completing a SESAME table to use for the subsequent hydrocode computations of section Section 4. It is easy to check that the extended transformation

$$L[V, \eta] : \begin{pmatrix} x \\ \\ y \\ \\ z \end{pmatrix} \mapsto \begin{pmatrix} y + \eta \\ \\ \boldsymbol{\nabla} V(y) - x \\ \\ z - xy + V(y) \end{pmatrix} \tag{9}$$

(where z represents the integral variable, i.e. the free energy) is invertible and a contact transformation, restricting to the symplectic transformation when projected onto the (x, y) variables. This can be used either as a post-processing step, or during training (where the generalization to a contact neural network is straight-forward) if integral variable data is available.

B.3 Manifold Losses

Aside from the classical pointwise distances between manifolds (ℓ^2 in the case of descrete samples), we also consider 'Manifold losses', which are Hausdorff-like distances between point clouds (that are permutation invariant). Any manifold $\mathcal{M}$ is only sampled by a finite collection of points, and we define our notions of distance accordingly, though there exist consistent formulations in the continuous setting.

Let $\{\mathbf{p}_i\}_{i=1}^N = \{(\mathbf{x}_i^p, \mathbf{y}_i^p)\}_{i=1}^N$, with $\mathbf{x}_i, \mathbf{y}_i \in \mathbb{R}^n$, be a sample of a Lagrangian submanifold $\Lambda \subset \mathbb{R}^{2n}$ (i.e., a discrete collection of points) and define $\{\mathbf{q}_i\}_{i=1}^N$ analogously for Λ'. It is useful to first define:

$$d(\mathbf{q}, \mathcal{M}) = \min_{\mathbf{p}\in\mathcal{M}} \|\mathbf{q} - \mathbf{p}\|_2^2 \tag{10}$$

$$d(\mathcal{M}, \mathcal{M}') = \max_{\mathbf{q}\in\mathcal{M}'} d(\mathbf{p}, \mathcal{M}) \tag{11}$$

Eqn. (10) is the classical point-manifold distance, while Eqn. (11) is a way of generalizing it to a distance between manifolds. Importantly, it is *asymmetric*. From an optimization perspective, we would like to work with continuously relaxed versions of the min and max functions that appear above. To that end we will use the 'smooth minimum' function s_a which is continuous and tends to min in the limit as $a \to \infty$:

$$s_a\big(\{\mathbf{p}_{i=1}^N\}\big) = \frac{\sum_{i=1}^N \mathbf{p}_i e^{-a\mathbf{p}_i}}{\sum_{i=1}^N e^{-a\mathbf{p}_i}} \tag{12}$$

We will directly replace the max with a sum (alternatively an average) to define an equivalent asymmetric manifold distance

$$d_{s_a}(\mathcal{M}, \mathcal{M}') = \sum_{i=1}^N s_a\Big(\big\{\|\mathbf{p}_i - \mathbf{q}_j\|_2\big\}_{j=1}^N\Big) \tag{13}$$

This allows us to define the following general loss functions that can be used as optimization criteria for the symplectic correction of Section 3.2, whose attributes we discuss below:

$$L_2: \quad L_2(\mathcal{M}, \mathcal{M}') = \frac{1}{N}\sum_{i=1}^N \|\mathbf{p}_i - \mathbf{q}_i\|_2^2 \tag{14}$$

$$L_H: \quad L_H(\mathcal{M}, \mathcal{M}') = \max\{d(\mathcal{M}, \mathcal{M}'), d(\mathcal{M}', \mathcal{M})\} \tag{15}$$

$$L_H^*: \quad L_H^*(\mathcal{M}, \mathcal{M}') = d_{s_a}(\mathcal{M}, \mathcal{M}') \tag{16}$$

$$L_{Hx}^*: \quad L_{Hx}^*(\mathcal{M}, \mathcal{M}') = d_{s_a}(\mathcal{M}, \mathcal{M}') + \frac{1}{N}\sum_{i=1}^N \|\mathbf{x}_i^p - \hat{\mathbf{x}}_i^q\|_2^2 \tag{17}$$

The L_2 loss (Eqn. (14)) maps points with the same index i between Λ and Λ'. When minimized, it yields a *good* correspondence between the two manifolds pointwise, but comes with two nontrivial drawbacks: (a) It is not invariant to permutations in one sample, so one must be certain of the point-to-point correspondence between the manifold samples before optimizing. This is unfortunate since any permutation of the manifold sample *ought to* define the same geometric object. (b) It does not allow movement of a potential discontinuity (kink), since the index i where a discontinuity would be mapped to is fixed, predefined by the point-to-point correspondence of manifold samples. The first hurdle is overcome by the more natural Hausdorff-like distance L_H (Eqn. (15)), which is invariant to permutations (at the cost of being more expensive to compute). It does not directly solve the discontinuity-movement issue.

To address both we consider the asymmetric (denoted by *) Hausdorff-like distances L_H^* (Eqn. (16)) and L_{Hx}^* (Eqn. (17)). Aside from the fact that they are differentiable through the modification of the min and max

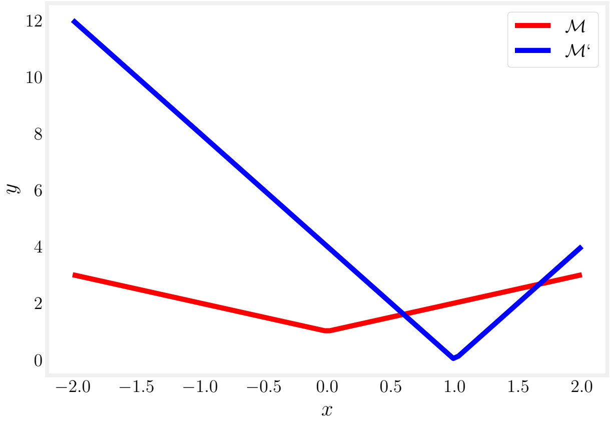

Figure 1: Graphical depiction of the piecewise smooth Lagrangian submanifolds Λ' (red) and Λ (blue).

functions of L_H, the removal of the symmetry constraint in L_H^* allows discontinuities to move more freely in ambient space. That comes at the cost of having small residuals even if points in Λ are far from points in Λ' (but not conversely). To counteract this effect, we introduce a Lagrangian stabilization term in L_{Hx}^* which, under a symplectomorphism $f : \Lambda \mapsto \Lambda'$ penalizes large changes in the Lagrangian subspace (where $\hat{x}_i^q$ is the x-coordinate of the image $f(\Lambda)$). This choice comes from the knowledge that $\mathbf{y}_i^p$ is ultimately a function of $\mathbf{x}_i^p$, so it is natural to ask for an adjustment of only the latter half of the coordinates, and this is further justified as a modelling choice in the context of Equations of State (EOS) (Appendix A.2) under the independent-variable assumption.

We note that the two individual terms of L_{Hx}^* may compete in their objectives: In their relative lexicographic limits, they effectively approximate L_H^* and L_2 respectively. However, our empirical results suggest that this competition can be critically useful when the terms are weighted appropriately. The following toy example offers some computational evidence of the preceding discussion.

Example B.1 (1D Toy)**.** Let $x \in [-2, 2]$ denote the independent variable, and consider two piecewise differentiable potential functions V and U given by:

$$V(x) = \frac{\operatorname{sgn}(x)x^2}{2} + x \tag{18}$$

$$U(x) = \frac{\operatorname{sgn}(x-1)(x-1)^2}{2} + 1 \tag{19}$$

Then, define the piecewise smooth Lagrangian submanifolds $\Lambda' = \{(x, v)\} \equiv \{(x, y) : y = V_x\}$ and $\Lambda = \{(x, u)\} \equiv \{(x, y) : y = U_x\}$ with the canonical two-form $\omega = \mathrm{d}x \wedge \mathrm{d}y$, which is well-defined everywhere except from a set of measure zero on each (Fig. 1).

We say that Λ approximates Λ' in the sense that they have the same topology; however the location of their discontinuity in the derivative (kink) and the *measure*[1] of each manifold on either side of it are different. To learn the correction between Λ and Λ', we sample each manifold using an equally spaced grid of 100 points in x, and train a symplectic network f_{NN} under the various loss functions listed in Appendix B.3. This simple example demonstrates the issues discussed there more abstractly.

In Fig. 2 we show snapshots of the training process of a symplectic network f_{NN} attempting to match Λ to Λ' under the L_{Hx}^* loss, at different training epochs t. At each t we observe that Λ' and $f_{\text{NN}}(\Lambda)$ have the same topology, i.e., both are smooth aside from one point where their derivative does not exist, denoted by $\star$. Remarkably, we see that under this loss the resulting network maps the original discontinuity of Λ close to the one of Λ' in a completely unsupervised manner (with an approximate error ℓ_2 error of 0.1!).

[1] whether discrete or Lebesgue, induced by a uniform measure on the independent variable $x \in [0, 2]$

This also implies the concentration of the sample points to the left of the discontinuity (due to the measure imbalance), and a spread of the ones to the right (due to the Lagrangian term of the loss).

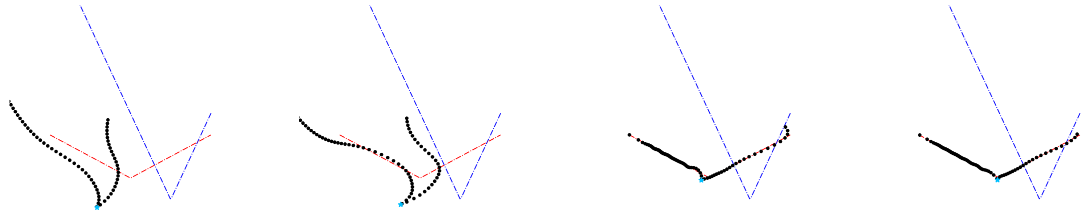

$t = 5 \times 20$ iterations $t = 10 \times 20$ iterations $t = 50 \times 20$ iterations $t = 106 \times 20$ iterations

Figure 2: Snapshots of training process under L^*_{Hx}. The input to the symplectic network is the manifold Λ (blue), while the output (black) is matched to the target manifold Λ' (red). The star ($\star$) denotes the image of the point where the discontinuity in the derivative occurs.

Fig. 3 depicts 'good' converged results after training the same symplectic network with different loss functions (L_2, L^*_H, L^*_{Hx}). We see that L_2 does not move the discontinuity at all while covering the full target manifold Λ'. Contrastingly, L^*_H correctly matches the discontinuities while leaving part of the target manifold (right side of Λ' in Fig. 3b) uncovered (which may imply extrapolation error). A good balance between the two behaviors is achieved by minimizing L^*_{Hx} (Fig. 3c). On an elementary level, this variety in the density of $f_{\text{NN}}(\Lambda)$ which allows both objectives to be satisfied in the latter case is directly caused by the competing terms in the L^*_{Hx} loss. (It is interesting to compare the result of minimizing of L^*_{Hx} in Fig. 2 and 3). In Table 1 we compare the numerical values of all losses after minimizing each individual one (denoted in the right column). The values across each line should be compared vertically and not horizontally, since they make use of different statistics when computing distances. Overall, we observe the numerical evidence of the preceding qualitative discussion: L_2 is competitive across all metrics aside from matching the discontinuity points of Λ and Λ', with a good middle ground in performance achieved by optimizing L^*_{Hx}.

At this point, we emphasise the use of the word 'good' in the previous paragraph. The optimization results (of e.g., Fig. 3) are sensitive to both the initialization of the network and the optimization procedure in the following manner: The asymmetric manifold distance is minimized when $f_{\text{NN}}(\Lambda)$ is in the interior of Λ', so in principle the black points can be folded to only fit on a small, non-representative part of Λ'. Increasing the weight of the Lagrangian term counteracts this issue, but comes at a cost of being less sensitive to topology. Additionally, large learning rates are capable of distorting ambient space very quickly and are hard to invert once a local minimum is achieved. This phenomenon can be reduced by using smaller learning rates. Importantly, the sole reason we are able to match the discontinuities of the two manifolds is that it is 'expensive' for our symplectic networks to create or destroy them. However, it is capable of doing so (as seen e.g., in Fig. 3a) and if that occurs, topological characteristics can then be lost (in the sense that they are no longer factored in by the loss).

Despite these issues, we note that that it is not unreasonable to mark and match discontinuities in a supervised manner, and to have a good approximation of $\mathcal{M}$ as an initial guess. That information, depending on the application, may often be tractably available, and considerably reduces the computational issues discussed.

B.4 Establishing Lipschitz Control

The previous example (Example B.1) demonstrates that coupling Hénon networks with a manifold loss can implicitly 'identify' and move discontinuities. Since the networks are diffeomorphisms, they are guaranteed to preserver the local differential structure of submanifolds, and will preserve a discontinuity of their input; however, they are capable of smoothing out such a discontinuity, and alternatively warping a smooth part of the input to approximate a discontinuity. This behavior is visible in Fig. 3, where, under the L_2 loss, the network is 'forced' to smooth out the area surrounding the kink.

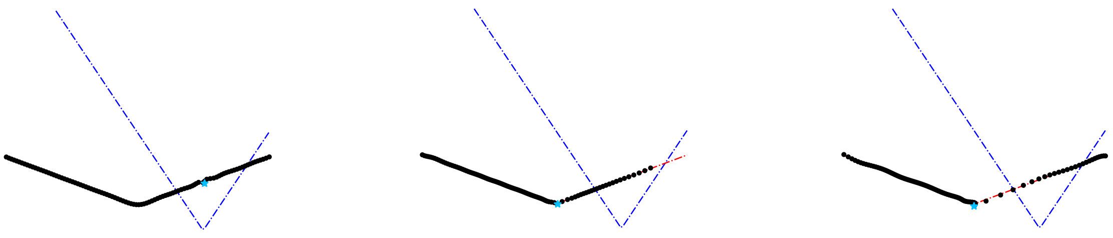

Figure 3: Mappings of $\mathcal{M}'$ (blue) to $\mathcal{M}$ (red) obtained using f_{NN} with different choices of loss function. The image of the point where the discontinuity occurs in $\mathcal{M}'$ is denoted by a star ($\star$).

Objective	L_2	L_H	L_H^*	L_{Hx}^*	Discontinuity
L_2	**2 e-4**	0.048	0.101	0.101	✗
L_H^*	0.585	0.132	**0.106**	0.815	✓
L_{Hx}^*	0.373	0.077	0.101	**0.568**	✓

Table 1: Loss comparison corresponding to Fig. 3. The optimized objective (left column) compared to the other loss values evaluated on the *test* set after convergence of the neural network. The right-most column qualitatively states if the discontinuity points of are matched.

In this section we demonstrate that Hénon networks are Lipschitz maps, and propose control of their Lipschitz constant to improve training stability. The Lipschitz constant is an additional meta parameter of the network and must be chosen appropriately for the problem.

Formal Statement

Proposition B.1. A Hénon map is (l, L)-bi-Lipschitz where l, L are constants depending on $\boldsymbol{\nabla} V$. That is, there are constants l, L that satisfy

$$\frac{1}{l} d_X \left(\begin{pmatrix} x' \\ y' \end{pmatrix}, \begin{pmatrix} x \\ y \end{pmatrix} \right) \leq d_Y \left(H[V,\eta] \begin{pmatrix} x' \\ y' \end{pmatrix}, H[V,\eta] \begin{pmatrix} x \\ y \end{pmatrix} \right) \leq L d_X \left(\begin{pmatrix} x' \\ y' \end{pmatrix}, \begin{pmatrix} x \\ y \end{pmatrix} \right),$$

for arbitrary metrics d_Y, d_X on $\mathbb{R}^n$, for which we will generally use $\|\cdot\|$ in place of the Euclidean norm $\|\cdot\|_2$.

Proof. Given $x, y, \eta \in \mathbb{R}^n$ and a (smooth) potential function $V : \mathbb{R}^n \to \mathbb{R}$, we defined

$$H[V,\eta] : \begin{pmatrix} x \\ y \end{pmatrix} \mapsto \begin{pmatrix} y + \eta \\ -x + \boldsymbol{\nabla} V(y) \end{pmatrix}$$

in Appendix B. We have that:

$$
\begin{aligned}
H[V,\eta]\begin{pmatrix} x \\ y \end{pmatrix} - H[V,\eta]\begin{pmatrix} x' \\ y' \end{pmatrix} &= \begin{pmatrix} y'-y \\ x-x'+\boldsymbol{\nabla}V(y')-\boldsymbol{\nabla}V(y) \end{pmatrix} \\
&= \begin{pmatrix} 0 & I \\ -I & G(y,y') \end{pmatrix}\begin{pmatrix} x'-x \\ y'-y \end{pmatrix}
\end{aligned}
$$

where $G(y,y') \in \mathbb{R}^{n\times n}$ is a matrix function that satisfies:

$$\boldsymbol{\nabla}V(y') - \boldsymbol{\nabla}V(y) = G(y,y')(y'-y) \tag{20}$$

Thus, L is given by

$$L \doteq \sup_{y,y'\in\mathbb{R}^n} \left\| \begin{pmatrix} 0 & I \\ -I & G(y,y') \end{pmatrix} \right\|$$

We can bound L in terms of the norm of G as follows: For arbitrary matrices $\mathcal{M} \in \mathbb{R}^{2n\times 2n}$, $\mathcal{G} \in \mathbb{R}^{n\times n}$ such that

$$\mathcal{M} = \begin{pmatrix} 0 & I \\ -I & \mathcal{G} \end{pmatrix} \tag{21}$$

the euclidean norm of $\mathcal{M}$ is bounded:

$$
\begin{aligned}
\|\mathcal{M}\|^2 = \sup_{\|x\|^2+\|y\|^2=1} \|\mathcal{M}\left(\begin{smallmatrix} x \\ y \end{smallmatrix}\right)\|^2 &= \sup_{\|x\|^2+\|y\|^2=1} \left\| \begin{pmatrix} y \\ \mathcal{G}y-x \end{pmatrix} \right\|^2 \\
&= \sup_{\|x\|^2+\|y\|^2=1} \left\{ \|y\|^2 + \|\mathcal{G}y-x\|^2 \right\} \\
&\leq \sup_{\|x\|^2+\|y\|^2=1} \left\{ \|x\|^2 + \|y\|^2 + \|\mathcal{G}\|^2\|y\|^2 \right\} \\
&\leq 1 + \|\mathcal{G}\|^2
\end{aligned}
$$

The above bound is tight since it is achieved when $x = 0, y = \arg\sup_{\|y\|=1} \|\mathcal{G}y\|$, where the operator norm is defined as $\|\mathcal{G}\| = \sup_{\|z\|=1} \|\mathcal{G}z\| \quad \text{for} \quad z \in \mathbb{R}^n$. Thus, the Lipschitz constant for a Hénon transformation is given by

$$L = 1 + \sup_{y,y'} \|G(y,y')\|, \tag{22}$$

provided that $\|G(y,y')\|$ is bounded.

In a similar fashion, we can find the Lipschitz constant, l, of the inverse map,

$$H^{-1}[V,\eta] : \begin{pmatrix} x \\ y \end{pmatrix} \mapsto \begin{pmatrix} \boldsymbol{\nabla}V(x-\eta) - y \\ x-\eta \end{pmatrix},$$

which can be shown to be $l = L$.

Overall, these results imply that as long as $\|G(y, y')\|$, which depends on $\boldsymbol{\nabla} V$, is bounded, The Hénon transformation is *bi-Lipschitz* with constants $L, l = L$.

An immediate consequence of the preceding proposition is that Hénon layers and networks, which consist of compositions of Hénon maps, are also bi-Lipschitz

Proposition B.2. Hénon layers and networks are bi-Lipschitz maps, with their constants dependent on $\boldsymbol{\nabla} V$, $\{\boldsymbol{\nabla} V_i\}_{i=1}^K$ respectively.

Numerical Implementation

The bi-Lipschitz Hénon map is implemented using a similar approach to the bi-Lipschitz affine transformation (bLAT) layer introduced in [10]. The function, $V : \mathbb{R}^n \to 1$ is represented as an arbitrary width neural network,

$$V(y) = c^T \theta(\mathcal{U}\Sigma\mathcal{V}^T y + b), \tag{23}$$

where $\mathcal{U} \in \mathbb{R}^{k\times r}, \Sigma \in \mathbb{R}^{r\times r}, \mathcal{V} \in \mathbb{R}^{n\times r}, b \in \mathbb{R}^k, c \in \mathbb{R}^k$, $r = \min(k, n)$, and θ is an element-wise activation function. We construct $\mathcal{U}, \Sigma, \mathcal{V}$ so that $A = \mathcal{U}\Sigma\mathcal{V}^T$ is a singular value decomposition. Therefore, $\mathcal{U}, \mathcal{V}$ have orthogonal columns and Σ is a diagonal matrix of singular values, $\Sigma = \mathrm{diag}(\sigma_1, \ldots, \sigma_r)$. Furthermore, we restrict the singular values $|\sigma_i| \leq \sigma_{\max}$ for some parameter $\sigma_{\max} > 0$. We additionally construct c such that the maximum element $\max_j |c_j| \leq c_{\max}$ for some parameter $c_{\max} > 0$.

Using index notation, the function V can be represented as

$$V(y) = \sum_i c_i \theta \left(\sum_j A_{ij} y_j + b_i \right). \tag{24}$$

The kth component of the gradient is given by

$$\frac{\partial}{\partial y_k} V(y) = \sum_i c_i A_{ik} \theta' \left(\sum_j A_{ij} y_j + b_i \right). \tag{25}$$

Let $z_i = \sum_j A_{ij} y_j + b_i$ and $z_i' = \sum_j A_{ij} y_j' + b_i$ Then

$$\begin{aligned} \frac{\partial}{\partial y_k} V(y') - \frac{\partial}{\partial y_k} V(y) &= \sum_i c_i A_{ik} \left(\theta'\left(z_i'\right) - \theta'\left(z_i\right) \right), \\ &= \sum_i c_i A_{ik} \lim_{\epsilon\to 0} \frac{\theta'\left(z_i'\right) - \theta'\left(z_i\right)}{z_i' - z_i + \epsilon} (z_i' - z_i), \\ &= \sum_i c_i A_{ik} \lim_{\epsilon\to 0} \frac{\theta'\left(z_i'\right) - \theta'\left(z_i\right)}{z_i' - z_i + \epsilon} \sum_j A_{ij}(y_j' - y_j). \end{aligned} \tag{26}$$

Therefore the (k, j) element of $G(y', y)$ is given by

$$G(y', y)\big|_{kj} = \sum_i c_i A_{ik} A_{ij} \lim_{\epsilon\to 0} \frac{\theta'\left(z_i'\right) - \theta'\left(z_i\right)}{z_i' - z_i + \epsilon}. \tag{27}$$

This can be written in matrix notation using

$$G(y', y) = A^T \mathrm{diag}(c) \, \mathrm{diag} \left(\lim_{\epsilon\to 0} \frac{\theta'\left(z_i'\right) - \theta'\left(z_i\right)}{z_i' - z_i + \epsilon} \right) A. \tag{28}$$

Here, $\text{diag}(c) = \begin{bmatrix} c_1 & \dots & c_k \end{bmatrix}^T$, etc. Therefore,

$$\|G(y', y)\|_2 \leq \kappa_{\theta'} \|A\|_2^2 \max_j |c_j|, \tag{29}$$

where

$$\kappa_{\theta'} := \sup_{z,z'} \lim_{\epsilon \to 0} \frac{|\theta'(z') - \theta'(z)|}{|z' - z + \epsilon|}. \tag{30}$$

Due to the mean value theorem,

$$\kappa_{\theta'} \leq \sup_z |\theta''(z)|. \tag{31}$$

This constant can be determined analytically or numerically for different choices of activation functions. For $\theta = \tanh$, we have $\kappa_{\theta'} = \frac{4}{3\sqrt{3}} \approx 0.7698$.

By construction, we have that $\|A\|_2 = \sigma_{\max}$ and $\max_j |c_j| = c_{\max}$. Therefore, the Lipschitz constant for the Hénon layer involving V satisfies

$$L = 1 + \kappa_{\theta'} \sigma_{\max}^2 c_{\max}. \tag{32}$$

We note that in our implementation of the layer, the orthogonal matrices are parameterized using the Householder factorization.

C Architectures and Data Sets

C.1 SESAME tables

In our numerical examples, we utilize tabular EoS data generated with OpenSesame. In particular, we use 'ground truth' tables for lead (Pb) and Copper (Cu) along with a perturbed table for lead.

OpenSesame is a sophisticated code system specifically designed for developing and interacting with the Los Alamos National Laboratory's SESAME Equation of State (EoS) libraries. These libraries serve as a comprehensive collection of thermodynamic properties of materials, detailed in tables that elucidate the behavior of materials under a vast range of conditions. Central to OpenSesame is the utilization of the Helmholtz free energy as the foundational thermodynamic variable. This choice enables the creation of EoSs that represent material behavior across extensive temperature and pressure ranges. The system is essential for producing EoSs that are both theoretically robust, integrating advanced models and computational techniques, and practically valuable, closely aligning with experimental data and detailed calculations.

OpenSesame employs a three-term decomposition approach to produce EoSs that are reasonably accurate over broad temperatures and pressures. This method decomposes the total Helmholtz free energy, $F(V, T)$, into three distinct components: First, the cold curve, $\phi_0(V)$, encapsulates the internal energy's relationship with volume, absent thermal contributions. Next, the nuclear model, $F_{\text{ion}}(V, T)$, extends the cold curve by incorporating thermal ionic contributions, capturing the thermal effects on the atomic nuclei. Finally, the electronic model, $F_{\text{el}}(V, T)$, addresses the electronic contributions to the free energy. This last component is particularly crucial for metals, plasmas, and certain high-pressure/temperature phases of materials, playing a key role in modeling compressibility, thermal expansion, and phase stability under extreme conditions.

By summing these components at constant density (ρ) and temperature (T), OpenSesame generates a detailed ρ, T grid. This grid forms the basis for evaluating all other thermodynamic quantities, providing a comprehensive and nuanced understanding of material behavior across diverse environments. The decomposition approach ensures that OpenSesame can generate accurate and thermodynamically consistent EoSs, catering to a wide spectrum of scientific and engineering applications.

Temperature	K	[131, 36426]
Volume	m^3/Mg	[0.016, 0.100]
Entropy	MJ/kgK	[117e-3, 4.04e-5]
Pressure	GPa	[2610, 1.67e-8]
Free Energy	MJ/kg	[11, -32]

Table 2: Observables, units, and value ranges (of the independent variables) used in the numerical simulations involving SESAME tables. The dependent variable ranges vary based on the data set.

To evaluate the ability of our machine learning approaches to accurately produce EoS Tables we have selected to examine a system in which Lead (Pb) is present. These materials incorporate a variety of phases and models to accurately describe their physical properties under different conditions.

Pb is characterized by three solid phases—Body-Centered Cubic (BCC), Face-Centered Cubic (FCC), and Hexagonal Close-Packed (HCP)—and one liquid phase. The solid phases utilize the finite-strain model for the cold curve, indicating the strain behavior under stress. The electronic contributions are calculated using the Thomas-Fermi-Dirac (TFD) model, which incorporates quantum mechanical effects of electrons. The nuclear model, giknuc, bridges Debye's low-temperature harmonic oscillations and an ideal gas's high-temperature behavior. The transition from solid to liquid phases employs the Lindemann melt model, capturing the melting process based on atomic vibrations. For the liquid phase, the models used are finite-strain for structural behavior, TFD for electronic aspects, and HighTLiq—a model designed for high temperature liquid phases in a multiphase approach.

A sensitivity study was conducted to identify the parameters most influential in deviating from the base EoS, focusing on the FCC phase for and Pb, while also determining the range of each individual parameter that would produce a valid EoS. The parameters identified for potential adjustments were the FCC `cold_bulk_modulus`, liquid `cold_dbdp`, and FCC `reference_gamma`. The `cold_bulk_modulus` measures resistance to compression, derived from the pressure-volume relationship at low temperatures. The `cold_dbdp` represents the pressure derivative of the bulk modulus at the reference density, indicating how the modulus changes with pressure. The `reference_gamma` is applied in the nuclear model to describe low-temperature behaviors.

With these parameters in mind, an ensemble of EoSs for Pb was generated through random sampling of the three identified parameters within the valid range. This approach allows for a comprehensive study of each parameter's impact on the material's properties. The initial, baseline parameters for Pb are [`cold_bulk_modulus`, `cold_dbdp`, and `reference_gamma`]= [46.903, 4.7, 2.6944]. Notably, the 73rd table for Pb was selected for further analysis in this work due to its significant deviations in hydrodynamic results when compared to the baseline EoS, with the values as a percentage of the base parameters $[134.61, 51.08, 143.75]\%$.

The tables list the values of $\{T_i, V_j, S_{ij}, P_{ij}, A_{ij}\}$ over a grid specified in the independent variables $\{T_i, V_j\}$. The range of the independent variables (T, V) considered in simulations is listed in Table 2, along with typical values of the dependent variables.

C.2 Admissible Regularization

Translations of data sets are admissible. However, we may not arbitrarily scale each individual variable since that distorts the symplectic structure of thermodynamic phase space Φ. Observe that for given coordinates of a symplectic manifold $\{x^1, x^2, y^1, y^2\}$, the following two scaling-group actions leave the symplectic form

invariant:

$$\{x^1, x^2, y^1, y^2\} \mapsto \left\{\alpha x^1, x^2, \frac{1}{\alpha} y^1, y^2\right\}, \quad \alpha \in \mathbb{R}^+$$
$$\{x^1, x^2, y^1, y^2\} \mapsto \left\{x^1, \beta x^2, y^1, \frac{1}{\beta} y^2\right\}, \quad \beta \in \mathbb{R}^+$$

while the following three change the symplectic form by a constant multiplicative factor:

$$\{x^1, x^2, y^1, y^2\} \mapsto \{\gamma x^1, \gamma x^2, y^1, y^2\}, \quad \gamma \in \mathbb{R}^+$$
$$\{x^1, x^2, y^1, y^2\} \mapsto \{x^1, x^2, \delta y^1, \delta y^2\}, \quad \delta \in \mathbb{R}^+$$
$$\{x^1, x^2, y^1, y^2\} \mapsto \{\zeta x^1, \zeta x^2, \zeta y^1, \zeta y^2\}, \quad \zeta \in \mathbb{R}^+$$

Thus, any combination of these transformations can be used to regularize thermodynamic data (which are of the form $\{T_i, V_i, S_i, P_i\}_{i=1}^N$ in our numerical experiments. In our computational examples, we typically use values between $(10^{-3}, 10^{-4})$ for α (multiplying temperature and entropy) and $(10^1, 10^2)$ for β (multiplying volume and pressure).

C.3 Computational Summary

We summarize each computational example in Table 3, where we note the type of model, objective optimized, and final discrepancy when optimization was stopped. The numerical values depend on the example, regularization, and the approximation parameter (a, as specified in Appendix B.3). Notably, the symplectic model in Section 4 does not perform as well as the extended model which also accounts for free energy values during training using the contact extension of Appendix B.2.

Example	Architecture	Optimized Loss	Final Loss
Example 3.1	Graph<br>Additive Graph	ℓ^2	3.57e-2<br>1.15e-2
Example 3.2	Symplectic	ℓ^2<br>L^*_{Hx}	0.27e-4<br>8.45e-6
Example 3.3	Symplectic	L^*_{Hx}	9.62e-1
Example 3.4	Symplectic	L^*_{Hx}	8.77e-3
Section 4	Additive Graph<br>Symplectic<br>Contact	ℓ^2<br>L^*_{Hx}<br>L^*_{Hx}	2.92e-2<br>1.23<br>1.11e-2

Table 3

C.4 Architecture Specifications

The mathematical description of the neural architectures used is described in Section 3. Here we specify the exact parameters by which they are implemented in each numerical example.

All networks are implemented in `python` using `pytorch` [11] and the default weight initialization is used. All (individual) network implementations are fully connected, with a combinaiton of tanh and linear activation

functions, as specified in Table 4. The networks are optimized using Adam [12]. All instances of Hénon networks were constructed by composing three Hénon layers.

Example	Estimated Function	Layers & Activation, Width
Example 3.1	$\hat{f}_G$	(4 tanh, 1 linear), 20
Example 3.1	$\hat{f}_G^+$	(4 tanh, 1 linear), 20
Example 3.2	$V_i(x)$	(2 tanh, 1 linear), 5
Example 3.3	$V_i(x)$	(2 tanh, 1 linear), 5
Example 3.4	$V_i(x)$	(2 tanh, 1 linear), 5
Section 4 (AGC)	$\hat{f}_G$	(4 tanh, 1 linear), 20
Section 4 (SC, ESC)	$V_i(x)$	(2 tanh, 1 linear), 20
Example B.1	$V_i(x)$	(2 tanh, 1 linear), 5

Table 4: Architecture specifications for each numerical example. Note that for $\hat{f}_G, \hat{f}_G^+$ the entire correction function is estimated by a fully connected neural network, while for the remaining examples with symplectic networks, only each component V_i of each Hénon map (Definition B.1) is estimated by a fully connected network.

C.5 Sampling of EoS

Throughout our work, we assume that we have a dense sample of the underlying input ('template') and target EoS. This sample need not be uniform. Because of the structural properties of the proposed methods, we guarantee that the output EoS that approximates the target is consistent with thermodynamics, and is in that sense 'physical'. However, given a finite data sample of the target, it is possible that several different EoS accurately interpolate it; in that sense, the output of each model may be one of several plausible physical ones that adhere to observations. The accuracy of the predicted EoS is thus not guaranteed away from the training data.

Similarly, if the location of a phase transition is not densely sampled, it may be hard to pinpoint its exact location. In that case, we are still guaranteed that the model features the right type of transition, but determining its 'true' location may not be possible.

D Hydrodynamic Simulation Details

To examine the ability of the machined learned EoS Tables to perform within the confines of a hydrodynamic simulation, a hydrodynamic test problem was constructed using the Los Alamos computational fluid dynamics program in which a series of Tantalum Shells with initial velocities given as depicted in Figure 5 explodes into a Lead and Copper System.

Pagosa is a 3D finite difference/volume Eulerian hydrodynamics program utilized for the study of high-pressure and high rate-rate deformation. .[13] A volume fluid equation is incorporated, due to the multi-material problems of interest. To close the the system of equations a EoS along with stress strain relationships are utilized. In this investigation we utilize Steinberg strength models for both Copper and Lead. .[14, 15]

Additional details of Pagosa include a Youngs Material Interface reconstruction algorithm to enable accurate shock capturing, a second order operator splitting algorithm, artificial viscosity, and a second order predictor corrector time integration methods.[13]

Lead table generation (Table 73)

OpenSesame is a code system for creating and interacting with equation of state (EOS) libraries such as the SESAME library produced by Los Alamos National Laboratory. OpenSesame divides the EOS into three main contributions: electronic, nuclear, and cold curve. Each contribution is calculated independently at various densities and temperatures and then summed to form the total EOS. The electronic contribution is typically modeled using Thomas-Fermi-Dirac (TFD) theory, with optional low-temperature interpolation for stability. The nuclear contribution approximates materials as a Debye solid at low temperatures and an ideal gas at high temperatures, with Grüneisen models to describe the zero-point contributions and specific heat characteristics. The cold curve defines the material's behavior at zero temperature and pressure, capturing reference energy and bulk modulus properties. Various models, such as finite strain or Hugoniot fits, can be used to describe the cold curve based on available data.

In our study, we varied three key parameters within the EOS of lead, starting from a baseline model (ground truth table ID: 320600) and creating a perturbed table (ID: 320673 or table 73). For the face-centered cubic (fcc) phase, we adjusted the cold_bulk_modulus in the finite strain model of the cold curve contribution and the reference_gamma parameter in the Debye model of the nuclear contribution. Additionally, we modified the cold_dbdp parameter (derivative of the bulk modulus with respect to pressure) in the finite strain model for the liquid phase of lead. The ground truth values for these parameters were 46.903 for the fcc cold_bulk_modulus, 2.6944 for the fcc reference_gamma, and 4.700 for the liquid phase cold_dbdp. These were perturbed to 63.136, 3.8732, and 2.4009, respectively, allowing us to explore how variations in these mechanical and thermodynamic properties influence the overall behavior of lead under extreme conditions.

References

[1] A. E. Mattsson, "Short introduction to relations between thermodynamic quantities," *Sandia National Laboratories, Albuquerque, NM, Report No. SAND*, vol. 2112, 2016.

[2] G. Jaeger, "The ehrenfest classification of phase transitions: Introduction and evolution," *Archive for history of exact sciences*, vol. 53, pp. 51–81, 1998.

[3] P.-W. Guan, "Differentiable thermodynamic modeling," *Scripta Materialia*, vol. 207, p. 114217, 2022.

[4] L. Younes, *Shapes and diffeomorphisms*, vol. 171. Springer, 2010.

[5] J. W. Burby, Q. Tang, and R. Maulik, "Fast neural poincaré maps for toroidal magnetic fields," *Plasma Physics and Controlled Fusion*, vol. 63, 12 2020.

[6] D. Turaev, "Polynomial approximations of symplectic dynamics and richness of chaos in non-hyperbolic area-preserving maps," *Nonlinearity*, vol. 16, pp. 123–135, nov 2002.

[7] G. Cybenko, "Approximation by superpositions of a sigmoidal function," *Mathematics of control, signals and systems*, vol. 2, no. 4, pp. 303–314, 1989.

[8] K. Hornik, M. Stinchcombe, and H. White, "Universal approximation of an unknown mapping and its derivatives using multilayer feedforward networks," *Neural networks*, vol. 3, no. 5, pp. 551–560, 1990.

[9] V. Duruisseaux, J. W. Burby, and Q. Tang, "Approximation of nearly-periodic symplectic maps via structure-preserving neural networks," *arXiv preprint arXiv:2210.05087*, 2022.

[10] D. A. Serino, A. A. Loya, J. Burby, I. G. Kevrekidis, and Q. Tang, "Intelligent attractors for singularly perturbed dynamical systems," *arXiv preprint arXiv:2402.15839*, 2024.

[11] A. Paszke, S. Gross, F. Massa, A. Lerer, J. Bradbury, G. Chanan, T. Killeen, Z. Lin, N. Gimelshein, L. Antiga, A. Desmaison, A. Kopf, E. Yang, Z. DeVito, M. Raison, A. Tejani, S. Chilamkurthy, B. Steiner, L. Fang, J. Bai, and S. Chintala, "Pytorch: An imperative style, high-performance deep learning library," in *Advances in Neural Information Processing Systems 32*, pp. 8024–8035, Curran Associates, Inc., 2019.

[12] D. P. Kingma and J. Ba, "Adam: A method for stochastic optimization," *arXiv preprint arXiv:1412.6980*, 2014.

[13] G. S. G. Subramanian, W. N. Weseloh, S. P. Clancy, and J. W. Painter, "Pagosa theory manual," tech. rep., Los Alamos National Lab.(LANL), Los Alamos, NM (United States), 2020.

[14] D. Steinberg and C. Lund, "A constitutive model for strain rates from 10- 4 to 106 s-," *Journal of applied physics*, vol. 65, no. 4, pp. 1528–1533, 1989.

[15] D. J. Steinberg, S. Cochran, and M. W. Guinan, "A constitutive model for metals applicable at high-strain rate," *Journal of applied physics*, vol. 51, no. 3, pp. 1498–1504, 1980.
